# Cadherin-7 enhances Sonic Hedgehog signalling by preventing Gli3 repressor formation during neural tube patterning

**DOI:** 10.1098/rsob.170225

**Published:** 2017-12-20

**Authors:** Rie Kawano, Kunimasa Ohta, Giuseppe Lupo

**Affiliations:** 1 Department of Medical Oncology and Hematology, Oita University Faculty of Medicine, Oita, Japan; 2 Global COE ‘Cell Fate Regulation Research and Education Unit’, Kumamoto University, Kumamoto, Japan; 3 Division of Developmental Neurobiology, Graduate School of Life Sciences, Kumamoto University, Kumamoto, Japan; 4 International Research Core for Stem Cell-based Developmental Medicine, Kumamoto University, Kumamoto, Japan; 5 Japan Agency for Medical Research and Development (AMED), Tokyo, Japan; 6 Department of Chemistry, Sapienza University of Rome, Rome, Italy

**Keywords:** Cadherin-7 (Cdh7), Sonic Hedgehog, suppressor-of-fused, Gli3, Gli3 repressor, neural tube patterning

## Abstract

Sonic Hedgehog (Shh) is a ventrally enriched morphogen controlling dorsoventral patterning of the neural tube. In the dorsal spinal cord, Gli3 protein bound to suppressor-of-fused (Sufu) is converted into Gli3 repressor (Gli3R), which inhibits Shh-target genes. Activation of Shh signalling prevents Gli3R formation, promoting neural tube ventralization. We show that cadherin-7 (Cdh7) expression in the intermediate spinal cord region is required to delimit the boundary between the ventral and the dorsal spinal cord. We demonstrate that Cdh7 functions as a receptor for Shh and enhances Shh signalling. Binding of Shh to Cdh7 promotes its aggregation on the cell membrane and association of Cdh7 with Gli3 and Sufu. These interactions prevent Gli3R formation and cause Gli3 protein degradation. We propose that Shh can act through Cdh7 to limit intracellular movement of Gli3 protein and production of Gli3R, thus eliciting more efficient activation of Gli-dependent signalling.

## 1. Introduction

The vertebrate neural tube is patterned along the dorsoventral (DV) axis to form different progenitor domains, identified by unique patterns of transcription factor expression and distinct neuronal fates [1]. This DV patterning results from a ventral-to-dorsal gradient of Sonic Hedgehog (Shh), which is complemented by bone morphogenetic proteins (BMPs) and Wnts acting in the dorsal neural tube. These morphogens provide positional information by promoting specific transcription factor profiles in each progenitor domain [2,3]. Although progenitor domains are sharply delimited along the DV axis, the mechanisms underlying well-defined boundaries of gene expression in the neural tube are still unclear.

Shh is secreted by the notochord and the neural tube floor plate, generating a dynamic ventral-to-dorsal gradient of concentration that results in spatio-temporally graded Shh pathway activation and ventral neural tube patterning [2,4,5]. Shh peptide is proteolytically cleaved to generate an N-terminal biologically active fragment (N-Shh) [6], which binds the transmembrane protein Patched1 (Ptch1) [7]. In the absence of N-Shh, Ptch1 represses the movement of another transmembrane protein, Smoothened (Smo), to the primary cilium [8]. When Ptch1 is bound to N-Shh, Smo can accumulate in the primary cilium and transduce Shh signalling intracellularly, modulating the activity of Gli1–3 transcription factors [6].

Gli2 and Gli3 exist as either full-length transcriptional activators (Gli2FL and Gli3FL) or truncated N-terminal fragments generated by partial proteolysis of the carboxyl terminus (Gli2R and Gli3R), acting as transcriptional repressors [9]. Suppressor-of-fused (Sufu) functions as an inhibitor of Shh signalling by binding Gli2/3FL proteins in the cytoplasm and preventing them from reaching the nucleus [10]. In the absence of Shh, Gli2/3FL proteins associated with Sufu are thought to be phosphorylated by PKA, GSK3β and CKI at the base of the primary cilium, causing their conversion into Gli2/3R [10–13]. Gli2R is completely degraded, but Gli3R translocates to the nucleus and represses transcription of Shh-target genes [14]. Activation of Shh signalling is believed to cause increased localization of unphosphorylated Gli2/3FL within the primary cilium, causing their transport to the nucleus, where they can activate Shh-target genes [10]. While Gli2FL is a strong transcriptional activator, Gli3FL has a low transcription-promoting activity and it rapidly undergoes proteosomal degradation when Shh signalling is active [11,12,15]. Therefore, GliR function can be mainly ascribed to Gli3R, while Shh-dependent transcriptional activation is mostly mediated by Gli2FL [10]. Despite major headway in dissecting the regulation of Shh signal transduction, there is still limited knowledge of the molecular mechanisms controlling trafficking and processing of Gli proteins.

Cdh7 is a member of the classical cadherin family, which is expressed in the intermediate neural tube region at the lower end of the Shh morphogen gradient [6]. Here, we show that Cdh7 is required to ventrally delimit the expression domain of dorsal spinal cord genes, thus allowing the specification of ventral territories. We provide evidence that Cdh7 exerts this function by positively regulating Shh signalling. The extracellular region of Cdh7 is bound by N-Shh, which alters Cdh7 distribution within the cell membrane. This promotes association of the intracellular domain of Cdh7 with Sufu and Gli3FL. As a result of these interactions, Gli3R production and nuclear accumulation are inhibited, and Gli3 protein is instead targeted for degradation, thus enhancing Shh signalling levels. These results reveal a new mechanism of Shh signalling regulation and help to understand how a highly dynamic spatio-temporal Shh gradient can result in well-defined boundaries of gene expression.

## 2. Results

### 2.1. Cdh7 expression in the intermediate spinal cord delimits Pax7-positive domain in the dorsal spinal cord

**Figure 1.**
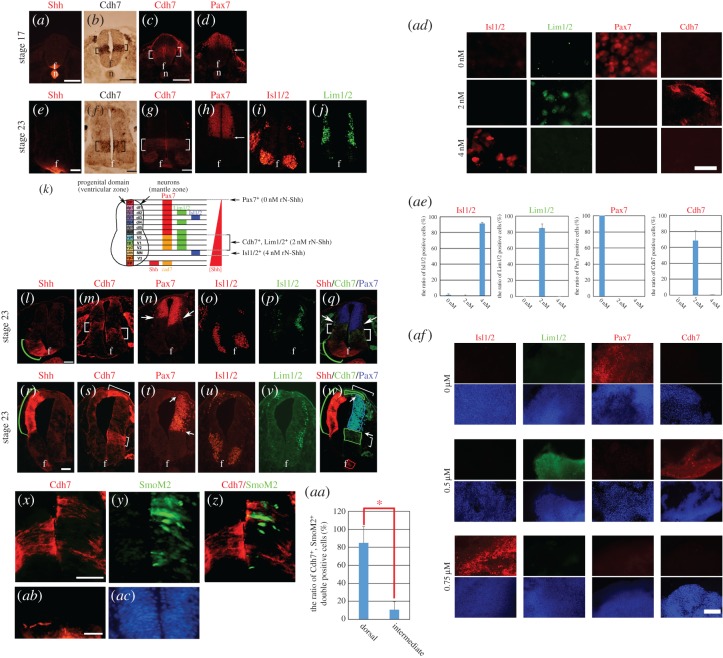
The concentration-dependent regulation of Cdh7 expression by Shh controls specification of the Pax7^+^/Pax7^−^ neural tube boundary. (*a–j*) Immunostaining (*a,c–e,g–j*) or *in situ* hybridization (*b,f*) analyses using anti-Shh mAb (*a,e*), *Cdh7* RNA probe (*b,f*), anti-Cdh7 mAb (*c,g*), anti-Pax7 mAb (*d,h*), anti-Isl1/2 mAb (*i*) or anti-Lim1/2 mAb (*j*) on transversal sections of HH st. 17 (*a–d*) or st. 23 (*e–j*) chick embryonic spinal cord. *Cdh7* mRNA and Cdh7 protein are detectable in the intermediate region of the developing neural tube (black and white brackets in (*b,f*) and (*c,g*), respectively), as well as in the floor plate (f) in (*f,g*). At HH st. 23, *Cdh7* transcription in the intermediate region is restricted to neural progenitors in the periventricular region (black brackets in (*f*), whereas Cdh7 protein is expressed throughout the neural tube wall (white brackets in (*g*)). The dorsal border of Cdh7 expression in the intermediate spinal cord abuts the ventral border of Pax7 expression in the dorsal spinal cord (cf. (*c,g*) with (*d,h*)) and coincides with the boundary between the dorsal and the ventral neural tube. At these stages, Shh is detectable in the neural tube floor plate (f) and the underlying notochord (n) (*a,e*). Isl1/2 is detectable in the dI3 and MN regions (*i*), and Lim1/2 is detectable in the dI2, dI4, dI6, V0, V1 and V2 regions (*j*) in HH st. 23 embryonic spinal cord; (*k*) shows a schematic diagram of the progenitor and neuronal domains and of the expression patterns of Shh, Cdh7, Pax7, Lim1/2 and Isl1/2 in the chick embryonic spinal cord. The ventral-to-dorsal gradient of Shh protein and the different Shh concentrations at which expression of Pax7, Cdh7, Lim1/2 and Isl1/2 is induced in spinal cord explants are shown on the right. See text for details: f, floor plate; n, notochord; Scale bars, 50 µm. (*l–w*) Adjacent transversal sections of HH st. 23 chick neural tube following unilateral electroporation of a chick Shh-expressing plasmid at HH st. 10 and immunostaining using anti-Shh (*l,r*), anti-Cdh7 (*m,s*), anti-Pax7 (*n,t*) anti-Isl1/2 (*o,u*) or anti-Lim1/2 (*p,v*) antibodies. The electroporated side is shown on the left; (*q,w*) shows merging of Shh (red), Cdh7 (green) and Pax7 (blue) staining as in (*l,r*), (*m,s*) and (*n,t*). Shh overexpression in the ventral neural tube causes coordinated dorsal shift of both Cdh7^+^ and Pax7^+^ domains. Shh overexpression in dorsal regions represses Cdh7 expression in the intermediate spinal cord and ectopically activates it in the roof of the neural tube. Green brackets in (*l,q,r,w*): ectopic Shh-expressing region. Red squares in (*w*); endogenous Shh-expressing region. White brackets in (*m,q,s,w*) and green squares in (*w*): endogenous and ectopic Cdh7-expressing regions. White arrows in (*n,q,t,w*) point to the boundaries between the Pax7^+^ and Pax7^−^ region. f, floor plate. Scale bar, 100 µm. (*x–ac*) Immunofluorescence analyses performed with anti-Cdh7 mAb (red signal, (*x,z*)), anti-GFP mAb (green signal, (*y,z*)) or anti-Shh mAb (red signal, (*ab*)) on transversal sections of HH st. 20 chick spinal cord tissue, following unilateral co-electroporation of plasmids expressing SmoM2 and EGFP at HH st. 10. The electroporated side is shown on the right. Quantification of the fraction of Cdh7^+^/EGFP^+^ cells in the dorsal and the intermediate spinal cord is shown in (*aa*). At least 10 sections from five different embryos were used for these analyses. Error bars represent s.e.m.; *p < 0.005 according to Student's *t*-test. SmoM2 expression causes Cdh7 upregulation in the dorsal spinal cord, but represses Cdh7 expression in the intermediate spinal cord. SmoM2 expression results in Shh upregulation within the ventral, but not the dorsal spinal cord (*ab*). Hoechst 33342 staining is shown in blue in (*ac*). Scale bars, 25 μm. (*ad*) Immunostaining using antibodies against Isl1/2, Lim1/2, Pax7 and Cdh7 on HH st. 10 explants of presumptive intermediate spinal cord, which were cultured for 20 h with 0, 2 or 4 nM rN-Shh. Scale bar, 100 µm. Quantification of the percentage of cells positive for the indicated markers is shown in (*ae*). At least each 500 cells were counted for these analyses. Error bars represent s.e.m. rN-Shh treatments cause Pax7 repression and dose-dependent upregulation of Cdh7-Lim1/2 (at 2 nM rN-Shh) or Isl1/2 (at 4 nM rN-Shh). (*af*) Immunostaining using antibodies against Isl1/2, Lim1/2, Pax7 and Cdh7 on HH st. 10 explants of presumptive intermediate spinal cord, which were cultured for 20 h with 0, 0.5 or 0.75 µM SAG. SAG treatments cause Pax7 repression and dose-dependent upregulation of Cdh7-Lim1/2 (0.5 µM SAG) or Isl1/2 (0.75 µM SAG). Scale bar, 100 µm.

In the developing chick spinal cord, at Hamburger & Hamilton (HH) [16] stage (st.) 17–23, Cdh7 is expressed in an intermediate domain confined to the Pax7-positive region in the dorsal spinal cord (figure 1*a–k*) [17–20]. Confirming previous observations [18], unilateral electroporation of *Shh*-encoding DNA in the ventral spinal cord of HH st. 10 chick embryos led to coordinated dorsal shift of both Pax7 ventral expression boundary and of Cdh7-positive domain (figure 1*l–q*). When Shh was overexpressed in the dorsal half of the spinal cord, the endogenous Cdh7 domain in the intermediate spinal cord was repressed, whereas ectopic Cdh7 expression was detectable in the most dorsal spinal cord region (figure 1*r–w*). Similar effects were detectable by overexpressing SmoM2, a constitutively active form of Smo [21]. Unilateral electroporation of *SmoM2*-encoding DNA in the spinal cord of HH st. 10 chick embryos led to ectopic upregulation of Cdh7 in SmoM2-expressing cells within the dorsal spinal cord, whereas endogenous Cdh7 expression was downregulated in SmoM2-expressing cells within the intermediate spinal cord (figure 1*x–aa*). Shh or SmoM2 overexpression in the intermediate spinal cord probably results in higher levels of Shh signalling in comparison with dorsal overexpression, due to additive effects with endogenous Shh signalling, and to the ability of SmoM2 to upregulate Shh expression in the ventral, but not in the dorsal spinal cord (figure 1*ab,ac*). Therefore, these results suggest that Cdh7 expression occurs within a specific range of Shh signalling activation.

This was confirmed by analysing Cdh7 expression, along with that of the ventral spinal cord marker Isl1/2, the intermediate marker Lim1/2 and the dorsal marker Pax7, in explants of presumptive intermediate neural tube that were cultured for 20 h with different concentrations of recombinant N-Shh (rN-Shh) (figure 1*ad,ae*). Isl1/2 expression was observed at 4 nM rN-Shh and Lim1/2 expression at 2 nM N-Shh, while Pax7 expression was repressed by rN-Shh. Cdh7-positive cells were detectable only in the presence of 2 nM rN-Shh, indicating that Cdh7 expression is specifically promoted by low/moderate doses of Shh signalling (figure 1*ad,ae*).

Similar results were obtained using SAG (3-chloro-N-[trans-4-(methylamino)cyclohexyl]-N-[3-(4-pyridinyl)benzyl]-1-benzothiophene-2-carboxamide), a small molecule agonist of Smo [22] (figure 1*af*).

**Figure 2.**
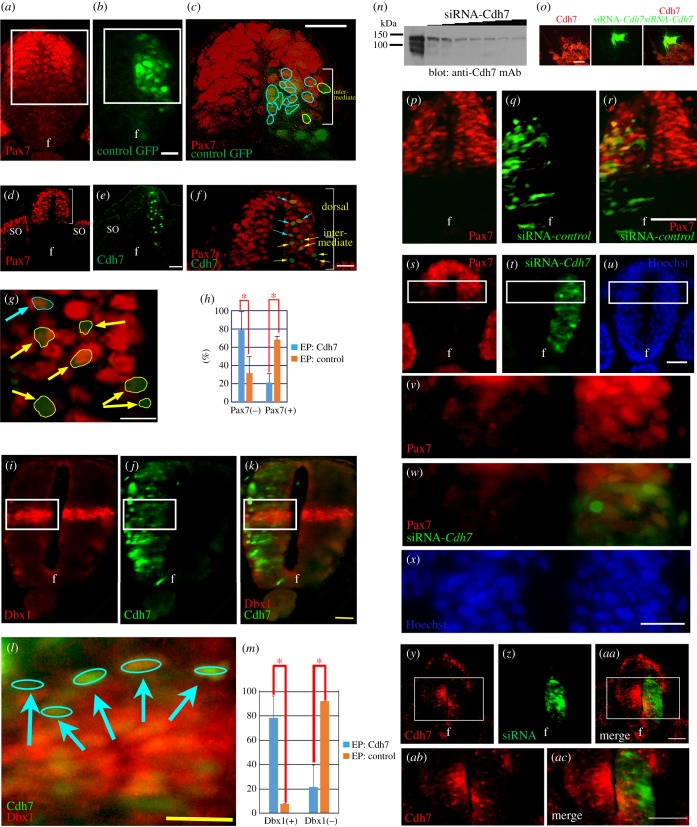
Shh-dependent Cdh7 expression controls specification of the Pax7^+^/Pax7^−^ neural tube boundary. (*a–c*) Immunostaining analysis with anti-Pax7 (red staining) and anti-GFP (green staining) mAbs on HH st. 15 chick neural tube, following unilateral electroporation of a control plasmid driving expression of a chimeric GFP protein fused to a nuclear localization signal; (*c*) shows a higher magnification of boxed regions in (*a*) and (*b*). Yellow and blue circles indicate Pax7-negative and Pax7-positive electroporated cells, respectively, within the intermediate spinal cord region (bracket). Within this region, most of the GFP-expressing cells are Pax7-positive. f, floor plate. Scale bars, 50 µm. (*d–g*) Immunostaining with anti-Pax7 (red staining) and anti-GFP (green staining) antibodies on HH st. 15 neural tube, following unilateral electroporation at HH st. 10 of a plasmid driving expression of both Cdh7 and a chimeric GFP protein fused to a nuclear localization signal; (*f*) shows a higher magnification of (*d,e*) at the level of the dorsal spinal cord, while (*g*) shows further enlargement of the electroporated intermediate spinal cord region. Cells overexpressing Cdh7 (green cells in (*e*), (*f*) and (*g*)) within the intermediate or the dorsal spinal cord are mostly negative or positive for Pax7, respectively. Brackets indicate the dorsal half of the spinal cord in (*d*), and the intermediate (bottom bracket) or the dorsal (top bracket) spinal cord regions in (*f*). In (*f,g*), yellow arrows point to electroporated cells that are Pax7^−^, whereas blue arrows point to electroporated Pax7^+^ cells. f, floor plate; so, somite. Scale bar, 100 µm for (*e*); 50 µm for *f*; 25 µm for (*g*). (*h*) Quantification of the percentage of the electroporated Pax7^−^ or Pax7^+^ cells within the intermediate spinal cord region, following electroporation of expression plasmids (EP) encoding for both Cdh7 and GFP (blue bars) or GFP only (orange bars). At least 13 sections from three different embryos were used for these analyses. Error bars represent s.e.m.; *p < 0.005 according to Student's *t*-test. (*i–l*) Immunostaining with anti-Dbx1 (red staining) and anti-GFP (green staining) antibodies on HH st. 15 neural tube, following unilateral electroporation at HH st. 10 of a plasmid driving expression of both Cdh7 and a chimeric GFP protein fused to a nuclear localization signal; (*l*) shows a higher magnification of the boxed area (*i,j,k*). Blue arrows point to electroporated Dbx1^+^ cells. Scale bar, 40 µm for (*k*); 20 µm for (*l*); (*m*) shows the percentage of the electroporated cells ectopically expressing Dbx1 following electroporation of EP encoding for both Cdh7 and GFP (blue bars) or GFP only (orange bars). At least 15 sections from five different embryos were used for these analyses. Error bars represent s.e.m.; *p < 0.005 according to Student's *t*-test. (*n,o*) siRNA-mediated knock-down of Cdh7 protein in L-Cdh7 cells with stable expression of Cdh7, as shown by immunoblotting (*n*) and immunostaining (*o*) analyses. In (*o*), cells electroporated with plasmid expressing siRNA-*Cdh7* and GFP are labelled in green, while red staining shows Cdh7 expression. Scale bar, 50 µm. (*p–r*) Immunostaining with anti-Pax7 (red staining, *p,r*) or anti-GFP (green staining, *q,r*) mAbs in transversal sections of HH st. 15 neural tube following unilateral co-electroporation of a plasmid encoding for siRNA-*control* and GFP at HH st. 10. The expression domain of Pax7 is not affected in the electroporated side. Scale bar, 50 µm. (*s–x*) Immunostaining with anti-Pax7 (red staining) or anti-GFP (green staining) antibodies on HH st. 15 neural tube following unilateral co-electroporation of a plasmid encoding for siRNA-*Cdh7* and GFP at HH st. 10. Images in (*v*), (*w*) and (*x*) show high magnifications of the boxed areas in (*s*), (*t*) and (*u*), respectively. Hoechst 33342 (blue) staining is shown in (*u,x*). Cdh7 knock-down causes ventral ectopic expression of Pax7 in siRNA-*Cdh7*/GFP-expressing cells within the intermediate spinal cord region. f, floor plate; so, somite. Scale bars, 50 µm for (*u*); 25 µm for (*x*). (*y–ac*) Immunostaining with anti-Cdh7 (red staining) or anti-GFP (green staining) mAbs on HH st. 15 chick neural tube following unilateral co-electroporation of a plasmid encoding for siRNA-*Cdh7* and for GFP at HH st. 10. Images in (*ab*) and (*ac*) show high magnifications of the boxed areas in (*y*) and (*aa*), respectively. In the electroporated side (shown on the right), Cdh7 expression is abrogated in GFP-positive cells. f, floor plate. Scale bars, 50 µm.

To gain more insight into Cdh7 function in the DV patterning of the neural tube, we electroporated a plasmid co-expressing green fluorescent protein (GFP) and Cdh7 into HH st. 10 chick spinal cord, and analysed its effects on Pax7 expression at HH st. 15. Control embryos were electroporated with a plasmid expressing GFP only. In control embryos, GFP-expressing cells located in the dorsal half of the spinal cord were mostly Pax7-positive (Pax7^+^) (figure 2*a–c*). By contrast, a significant fraction of the cells overexpressing Cdh7 were Pax7-negative (Pax7^−^) (figure 2*d–g*; yellow-labelled and blue-labelled cells or arrows in figure 2*c,f,g* correspond to Pax7^−^ and Pax7^+^-electroporated cells, respectively). When Pax7^+^ cells were counted in the intermediate neural tube close to the ventral border of Pax7 expression domain, a clear reduction in the fraction of Pax7^+^ cells was observed in Cdh7-expressing cells in comparison with cells expressing GFP only (figure 2*h*; electronic supplementary material, figure S1*a–n*). Dorsally localized Cdh7-overexpressing cells, however, retained Pax7 expression (figure 2*g*; electronic supplementary material, figure S1*g–n*). By staining electroporated embryos with an antibody against Dbx1, a marker of the p0 progenitor domain [23], we found ectopic Dbx1^+^ cells close to the dorsal border of the Dbx1 expression domain upon Cdh7 overexpression, but not in embryos expressing GFP only (figure 2*i–m*; electronic supplementary material, figure S1*o–ad*). Thus, Cdh7-dependent signalling can repress Pax7 expression specifically in the intermediated spinal cord region, leading to the acquisition of more ventral fates.

To test whether Cdh7 is required for proper spinal cord patterning, we electroporated HH st. 10 embryos with a plasmid expressing a *Cdh7*-targeting siRNA (siRNA-*Cdh7*), which could effectively knock down Cdh7 expression (figure 2*n,o,y–ac*), or with a control siRNA plasmid (siRNA-*control*). Electroporation of siRNA-*Cdh7*, but not of siRNA-*control*, caused ventral expansion of the Pax7^+^ domain (figure 2*p–x*).

Taken together, these data suggest that Shh-dependent expression of Cdh7 in the intermediate spinal cord is key to define the ventral boundary of the Pax7-expressing domain in the dorsal spinal cord.

### 2.2. Cdh7 promotes Shh signalling by binding Gli3FL and Sufu with a Shh-dependent mechanism

We analysed whether Cdh7 can modulate Shh signalling by means of reporter assays with a Shh-sensitive Gli-binding site–luciferase reporter plasmid (GBS-luc reporter [15]) on chick neural plate explants. We electroporated a plasmid mixture including a Cdh7 expression vector, the GBS-luc reporter plasmid and an internal control plasmid into HH st. 10 embryonic spinal cord. After 6 h, explants of the presumptive intermediate spinal cord were dissected and incubated for 20 h with or without rN-Shh before assaying for relative luciferase activity (RLA), as described in the electronic supplementary material, figure S2.

**Figure 3.**
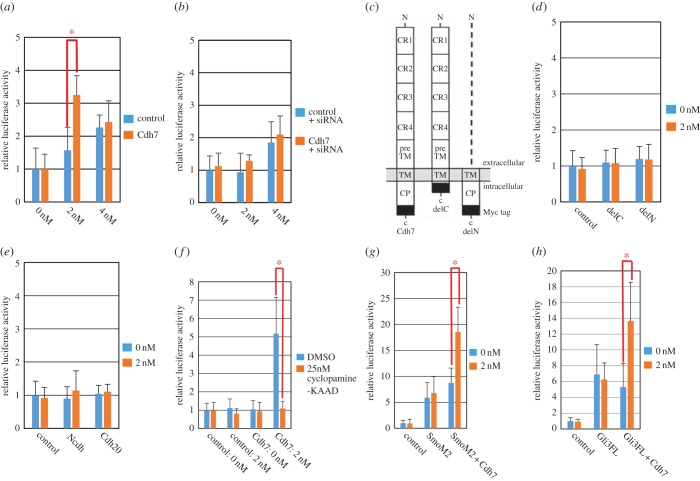
Cdh7 enhances Shh signalling by acting at the level of Gli3FL. (*a*) RLA quantification in HH st. 10 explants of presumptive intermediate spinal cord, which were dissected from embryos electroporated with GFP (control) or Cdh7 expression plasmids along with GBS-luc reporter and internal control plasmids, and treated with the indicated doses of rN-Shh as described in the electronic supplementary material, Material and methods. Cdh7 overexpression significantly enhances RLA in the presence of 2 nM rN-Shh, but not in untreated explants or in the presence of 4 nM rN-Shh. Results are shown as the mean of more than 12 independent experiments. Error bars represent s.e.m.; *p < 0.005 according to Student's *t*-test. (*b*) RLA quantification in explants that were treated as in (*a*) but also electroporated with siRNA-*Cdh7*. Cdh7 knock-down abolishes the effects of Cdh7 overexpression. (*c*) Schematic diagram of Myc-tagged Cdh7 full-length and two deletion constructs (delC and delN) as used for the assays shown in (*d*). (*d*) RLA quantification in explants that were treated as in (*a*) but electroporated with Cdh7 deletion constructs in place of full-length Cdh7. Cdh7 requires both its extracellular and intracellular regions to enhance Shh signalling. (*e*) RLA quantification in explants that were treated as in (*a*) but electroporated with constructs encoding for Ncdh or Cdh20 instead of Cdh7. Cdh7 ability to enhance Shh signalling is not shared by other cadherins. (*f*) RLA quantification in explants that were treated as in (*a*) but also exposed to DMSO or 25 nM cyclopamine-KAAD. Smo inhibition abrogates the ability of Cdh7 to enhance Shh signalling. (*g*) RLA quantification in explants that were treated as in (*a*) but electroporated with either SmoM2 or both SmoM2 and Cdh7 expression constructs. Cdh7 can further enhance Shh signalling in the presence of both SmoM2 and 2 nM rN-Shh. (*h*) RLA quantification in explants that were treated as in (*a*) but electroporated with either Gli3FL or both Gli3FL and Cdh7 expression constructs. Cdh7 can enhance Shh signalling in combination with both Gli3FL and 2 nM rN-Shh.

In these assays, treatments with 4 nM rN-Shh caused significant RLA increase, indicating upregulation of Shh signalling, whereas the effects of 2 nM rN-Shh were not significant (figure 3*a*; *p*-values for these experiments are reported in the electronic supplementary material, table S1). Notably, Cdh7 overexpression significantly enhanced Shh signalling in the presence of 2 nM rN-Shh, but not in the presence of 4 nM rN-Shh or in the absence of rN-Shh (figure 3*a*; electronic supplementary material, table S1). The specificity of this effect was confirmed by co-electroporation of plasmids expressing Cdh7 and siRNA-*Cdh7* (figure 2*n,o*), which abolished the increase in reporter expression observed in Cdh7-expressing explants (figure 3*b*; electronic supplementary material, table S1). Furthermore, reporter expression was not enhanced upon overexpression of truncated versions of Cdh7 lacking the intracellular or the extracellular domains, respectively (figure 3*c,d*; electronic supplementary material, table S1). N-cadherin (Ncdh) and cadherin-20 (Cdh20) were unable to enhance Shh signalling in this assay (figure 3*e*; electronic supplementary material, table S1). These observations suggest that Cdh7 is a specific binding partner of Shh and a positive regulator of Shh signalling in the presence of low/moderate levels of Shh ligand.

We investigated the mechanisms of Cdh7-dependent regulation of Shh signalling in more detail. We found that 25 nM cyclopamine-KAAD, a specific Smo inhibitor [21], abrogated Cdh7-dependent upregulation of GBS-luc reporter expression (figure 3*f*; electronic supplementary material, table S1), indicating that Cdh7 requires Smo function to promote Shh signalling. In agreement with these results, Cdh7 was able to reinforce GBS-luc reporter expression when overexpressed together with a constitutively active form of Smo (SmoM2) [21], especially in the presence of 2 nM rN-Shh (figure 3*g*; electronic supplementary material, table S1). We then analysed whether Cdh7 could exert similar effects following co-overexpression with the Smo effectors Gli1, Gli2 and Gli3. Notably, Cdh7 could enhance reporter expression in combination with 2 nM rN-Shh and Gli3FL (figure 3*h*; electronic supplementary material, table S1), but not Gli1, Gli2 or Gli3R (electronic supplementary material, figure S3; all *p*-values for the experiments shown in figure S3 are reported in electronic supplementary material, table S2). Similar results were obtained when performing GBS-luc reporter assays *in vitro* using NIH3T3 cells, which bear a functional Shh signalling pathway [24]. Confirming assays performed with chick embryo explants, GBS-luc reporter activity was significantly increased by 4 nM rN-Shh, but not by 2 nM rN-Shh. Furthermore, Cdh7 expression in NIH3T3 cells significantly enhanced Shh signalling together with 2 nM rN-Shh, but not with 4 nM rN-Shh or without rN-Shh. The effects of Cdh7 could not be mimicked by Cdh20 or deletion mutants of Cdh7, and they were sentitive to the presence of siRNA-*Cdh7* and KAAD-cyclopamine. Moreover, in the presence of 2 nM rN-Shh, Cdh7 could strengthen Shh signalling in collaboration with SmoM2 or Gli3FL, but not with Gli1, Gli2 and Gli3R (electronic supplementary material, figure S4; all *p*-values for the experiments shown in figure S4 are reported in electronic supplementary material, table S3). These results suggest that Cdh7 modulates Shh signalling by acting at the level of Gli3FL, but not through Gli1, Gli2 or Gli3R. They also suggest that Smo-dependent signal transduction is necessary for Cdh7 action and facilitates it.

Endogenously produced N-Shh peptide undergoes post-translational modifications through covalent binding of a cholesterol moiety at its C-terminus and a palmitate moiety at its N-terminus [10]. To confirm whether Cdh7 could functionally interact with fully lipidated N-Shh, we performed GBS-luc reporter assays with explants from chick embryos co-electroporated with plasmids encoding for Cdh7 and full-length chick Shh (cShh), instead of explant treatments with rN-Shh. The results of these assays generally showed higher experimental variability in comparison with rN-Shh treatments, probably due to variability in transfection efficiency of the cShh plasmid. Nonetheless, they largely recapitulated those obtained with rN-Shh, suggesting that Cdh7 can functionally interact with the fully lipidated N-Shh peptide (electronic supplementary material, figure S5; all *p*-values for the experiments shown in figure S5 are reported in electronic supplementary material, table S4).

### 2.3. Cdh7 binds Shh protein and enhances Shh signalling

**Figure 4.**
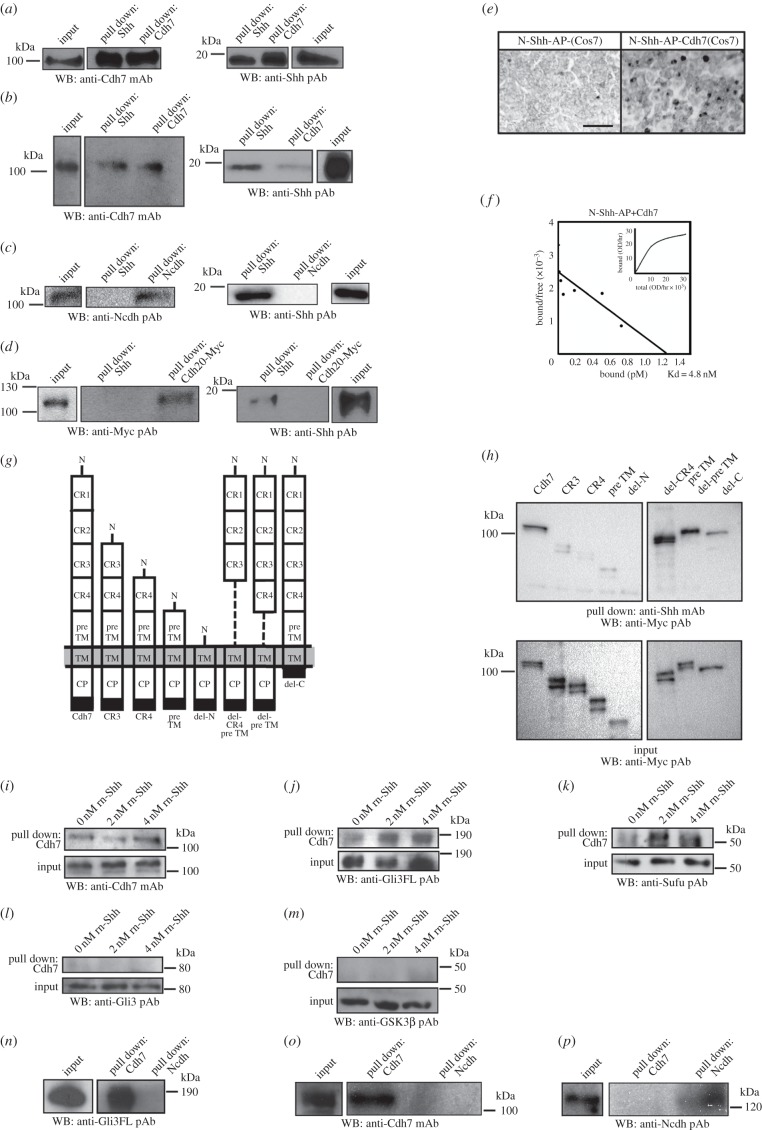
Cdh7 binds Shh and associates with Gli3FL and Sufu. (*a*) Immunoblotting with anti-Cdh7 mAb (left blot) or anti-Shh pAb (right blot) showing immunoprecipitation assays with lysates of NIH3T3 cells transiently expressing chick Cdh7 that were incubated with rN-Shh, followed by pull-down with anti-Shh or anti-Cdh7 mAbs. (*b*) Immunoblotting with anti-Cdh7 mAb (left blot) or anti-Shh pAb (right blot) showing the results of immunoprecipitation assays with HH st. 17 chick embryo lysates, following pull-down with anti-Shh or anti-Cdh7 mAbs. Both anti-Shh and anti-Cdh7 mAbs can co-immunoprecipitate both N-Shh and Cdh7. (*c*) Immunoblotting with anti-Ncdh pAb (left blot) or anti-Shh pAb (right blot) showing immunoprecipitation assays with lysates of NIH3T3 cells transiently expressing chick Ncdh that were incubated with rN-Shh, followed by pull-down with anti-Shh or anti-Ncdh mAbs. No co-immunoprecipitation of N-Shh and Ncdh is detectable. (*d*) Immunoblotting with anti-Myc pAb (left blot) or anti-Shh pAb (right blot) showing immunoprecipitation assays with lysates of NIH3T3 cells transiently expressing Myc-tagged chick Cdh20 that were incubated with rN-Shh, followed by pull-down with anti-Shh or anti-Myc mAbs. No co-immunoprecipitation of N-Shh and Cdh20 is detectable. (*e*) AP staining of control (left) or Cdh7-expressing (right) COS-7 cells that were incubated with conditioned medium from 293 cells expressing N-Shh-AP. N-Shh-AP signal is detectable only in Cdh7-expressing cells. Scale bar, 200 µm. (*f*) Saturation binding curve (inset) and Scatchard analysis of N-Shh-AP binding to Cdh7, showing a dissociation constant (Kd) of 4.8 nM. (*g*) Schematic diagram of Myc-tagged Cdh7 full-length and deletion constructs as used for the assays shown in (*h*). (*h*) Immunoblotting with anti-Myc pAb showing immunoprecipitation assays with lysates from COS-7 cells transiently expressing Cdh7 constructs shown in (*f*). Lysates were incubated with rN-Shh, followed by pull-down with anti-Shh mAb. Only constructs containing CR1 and CR2 domains are pulled down by anti-Shh mAb. The blot at the bottom shows the expression levels of each Cdh7 construct in lysates used for immunoprecipitation (input). (*i–m*) Immunoblotting with anti-Cdh7 mAb (*i*), anti-Gli3FL pAb (*j*), anti-Sufu pAb (*k*), anti-Gli3 pAb (*l*) or anti-GSK3β pAb (*m*) showing immunoprecipitation assays with NIH3T3 cells transiently expressing Cdh7 that were incubated with the indicated doses of rN-Shh, followed by pull-down with anti-Cdh7 mAb. Following cell treatment with rN-Shh, anti-Cdh7 mAb can co-immunoprecipitate Gli3FL and Sufu along with Cdh7, but not Gli3R and GSK3β. Note that anti-Gli3FL pAb used for (*j*) does not react with Gli3R, while anti-Gli3 pAb used for (*l*) reacts with both Gli3FL and Gli3R, and only the Gli3R 80 kDa band is shown. (*n–p*) Immunoblotting with anti-Gli3FL pAb (*n*), anti-Cdh7 mAb (*o*) or anti-Ncdh pAb (*p*) showing immunoprecipitation assays with HH st. 17 spinal cord lysates using anti-Cdh7 or anti-Ncdh mAbs. Anti-Cdh7 mAb, but not anti-Ncdh mAb, can co-immunoprecipitate both Gli3FL (*n*) and Cdh7 (*o*).

As shown above, Cdh7 can repress Pax7 expression in the intermediate spinal cord region, but not in more dorsal areas. We speculated that this spatially limited activity might be due to a molecular interaction between Cdh7 and Shh, which is not present in the dorsal spinal cord, and explored this possibility by means of co-immunoprecipitation assays. As described in the Material and methods (see electronic supplementary material), lysates of NIH3T3 cells transiently expressing chick Cdh7 were incubated with rN-Shh, followed by immunoprecipitation using an anti-Shh monoclonal antibody (mAb) or an anti-Cdh7 monoclonal antibody (mAb). Cdh7 (106 kDa) co-precipitated with N-Shh and vice versa (figure 4*a*), indicating direct binding between Cdh7 and N-Shh. N-Shh was not precipitated in pull-down experiments with anti-Cdh7 mAb using control NIH3T3 cells, which do not express Cdh7 (data not shown). Co-immunoprecipitation of endogenous N-Shh and Cdh7 was also observed using extracts from HH st. 17 spinal cord tissue (figure 4*b*). By contrast, rN-Shh did not co-immunoprecipitate with Ncdh or Cdh20 in assays with NIH3T3 cells transiently expressing Ncdh or Cdh20. (figure 4*c,d*). Both Ncdh and Cdh20 are expressed in the developing spinal cord [25,26], and Cdh20 is the closest cadherin family member to Cdh7 [27], indicating that the binding between N-Shh and Cdh7 is highly specific.

We performed saturation binding experiments using COS-7 cells expressing Cdh7, which were treated with conditioned medium from 293 cells expressing N-Shh tagged with alkaline phosphatase (AP) (figure 4*e*; electronic supplementary material, figure S6*a,b*). This analysis showed that the dissociation constant (Kd) between N-Shh and Cdh7 was approximately 4.8 nM (figure 4*f*). Ncdh, however, did not bind AP-tagged N-Shh (electronic supplementary material, figure S6*c,d*). By performing immunoprecipitation assays with different Cdh7 deletion mutants, we found that cadherin repeat 1 (CR1) and cadherin repeat 2 (CR2) domains are the major binding sites for N-Shh (figure 4*g,h*). Binding between N-Shh and Cdh7 was not prevented by anti-Shh mAb (clone 5E1), which inhibits N-Shh binding to Ptch1 (electronic supplementary material, figure S7) [28], suggesting that Shh interacts with Cdh7 and Ptch1 through different domains.

Sufu plays a pivotal role in controlling Gli3 processing to Gli3R [9,10]. Remarkably, co-immunoprecipitation assays using anti-Cdh7 mAb and Cdh7-expressing NIH3T3 cells showed stronger association of Cdh7 with Gli3FL and Sufu in cells cultured in the presence of rN-Shh, in comparison with control cells. In particular, treatments with 2 nM rN-Shh roughly doubled the amount of Gli3FL and Sufu bound to Cdh7 in comparison with untreated cells (figure 4*i–k*; western blot quantifications are reported in electronic supplementary material, table S5). By contrast, Cdh7 did not interact with Gli3R (figure 4*l*; electronic supplementary material, table S5) or GSK-3β neither in the absence nor in the presence of rN-Shh (figure 4*m*; electronic supplementary material, table S5). Co-immunoprecipitation assays with anti-Cdh7 or anti-Ncdh mAbs using HH st. 17 chick embryos confirmed that Cdh7, but not Ncdh, interacts with Gli3FL *in vivo* (figure 4*n–p*). These results suggest that Cdh7 can bind Gli3FL and Sufu at the intracellular level and that these interactions are strengthened when the extracellular region of Cdh7 is bound to Shh.

### 2.4. Shh promotes Cdh7 aggregation within the cell membrane

**Figure 5.**
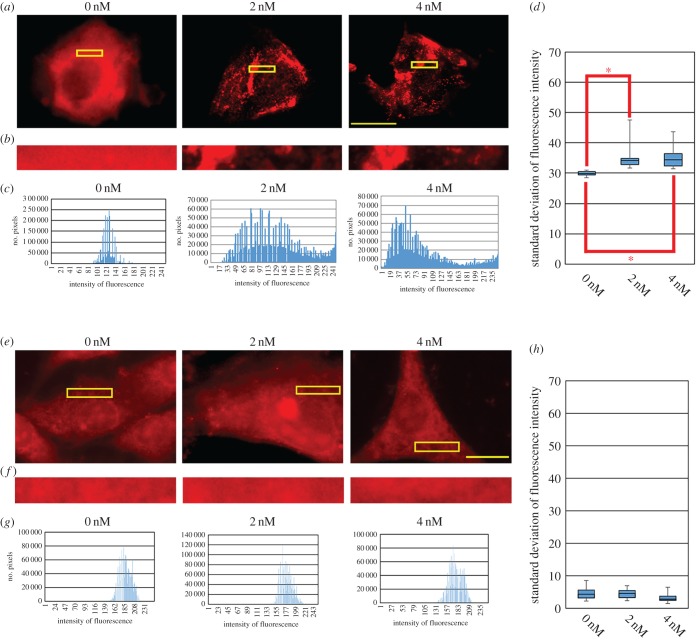

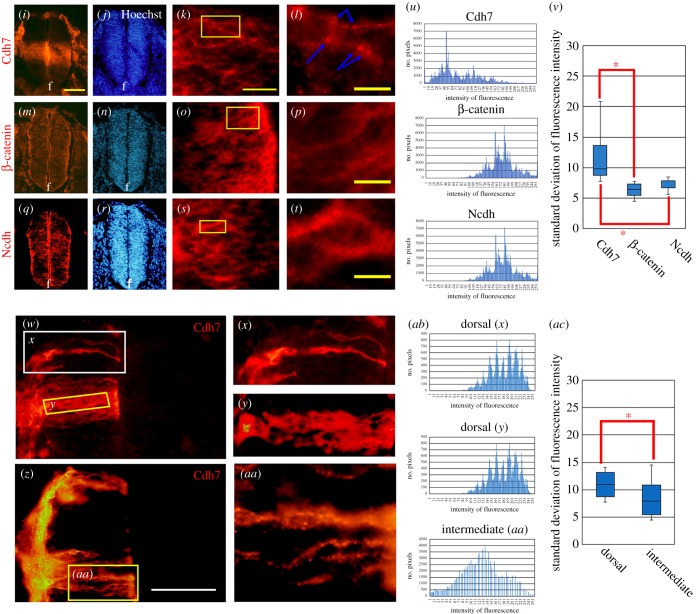
Shh promotes aggregation of Cdh7. (*a–d*) Immunofluorescence analysis performed with anti-Cdh7 mAb (red signal) using NIH3T3 cells transiently expressing Cdh7 that were cultured for 24 h in the indicated concentrations of rN-Shh. rN-Shh promotes Cdh7 aggregation at the cell membrane. Scale bar, 10 µm. (*b*) High magnifications of the yellow boxed areas in (*a*). (*c*) Distribution of fluorescence intensities in the representative images shown in (*b*) of Cdh7-expressing cells cultured without or with 2 nM or 4 nM rN-Shh. Charts report the number of pixels falling within a given range of fluorescence intensity. Cdh7 signal varies much more broadly in rN-Shh-treated samples in comparison with the untreated sample. (*d*) Box-and-whisker plots of the standard deviation of fluorescence intensities in Cdh7-expressing cells treated with the indicated concentrations of rN-Shh. Standard deviation of Cdh7 signal is significantly higher in rN-Shh-treated cells in comparison with control samples. Whiskers represent the distribution of the standard values for the fluorescence intensity of each image. The lower and higher whiskers indicate the minimum and maximum values, respectively. The bottom and top of the box represent the first and third quartiles, respectively, and the band inside the box indicates the second quartile (the median). At least 15 representative cells for each experimental condition were used for this analysis; *p < 0.005 according to Student's *t*-test. (*e–h*) Immunofluorescence analysis performed with anti-Ncdh mAb (red signal) using NIH3T3 cells transiently expressing Ncdh that were cultured for 24 h in the indicated concentrations of rN-Shh. rN-Shh treatments do not cause Ncdh aggregation at the cell membrane. (*f*) High magnifications of the yellow boxed areas in (*e*). (*g*) Distribution of fluorescence intensities in the representative images shown in (*e*) of Ncdh-expressing cells cultured without or with 2 nM or 4 nM rN-Shh. Charts report the number of pixels falling within a given range of fluorescence intensity. Ncdh signal spans a similar range of intensities in rN-Shh-treated samples in comparison with the untreated sample. (*h*) Box-and-whisker plots of the standard deviation of fluorescence intensities in Ncdh-expressing cells treated with the indicated concentrations of rN-Shh. Standard deviation of Ncdh signal is not significantly different in rN-Shh-treated cells in comparison with control samples. (*i–v*) Immunofluorescence analyses performed with anti-Cdh7 Ab ((*i–l*), red signal), anti-β-catenin Ab ((*m–p*), red signal) or anti-Ncdh Ab ((*q–t*), red signal) on transversal sections of HH st. 20 chick spinal cord tissue, showing aggregation of Cdh7 (blue arrows in (*l*)), but not β-catenin (*p*) or Ncdh (*t*), in cells within the intermediate spinal cord region. (*k,o,s*) show high magnification images of (*i,m,q*), respectively; (*l,p,t*) show high magnification images of the yellow boxed regions in (*k,o,s*), respectively. Hoechst 33342 staining is shown in blue in (*j,n,r*). Scale bar, 100 µm in (*i*), 50 µm in (*k*) and 10 µm in (*l,p,t*). (*u,v*) Distribution of fluorescence intensities in the representative images shown in (*l,p,t*). (*v*) Box-and-whisker plots of the standard deviation of fluorescence intensities in (*l,p,t*). Standard deviation of Cdh7 signal is significantly higher than that of β-catenin or Ncdh signal; *p < 0.005 according to Student's *t*-test. (*w–ac*) Immunofluorescence analysis performed with anti-Cdh7 mAb (red signal) on transversal sections of HH st. 23 chick spinal cord, which shows ectopic expression of Cdh7 in the dorsal neural tube following unilateral electroporation of a Cdh7-expressing construct at HH st. 10. The electroporated side is shown on the left. Lower levels of Cdh7 aggregates are detectable in dorsal spinal cord cells within ectopic Cdh7 expression (*w,x,y*) in comparison with Cdh7 aggregates present in the intermediate spinal cord (*z,aa*); (*x,y,aa*) show high magnification images of the boxed regions in (*w,z*) as indicated. Scale bar, 50 µm. (*ab*) Distribution of fluorescence intensities in the representative images shown (*x,y,aa*). (*ac*) Box-and-whisker plots of the standard deviation of fluorescence intensities in (*x,y,aa*). Standard deviation of Cdh7 signal is significantly higher in the intermediate than in the dorsal spinal cord region; *p < 0.005 according to Student's *t*-test.

As Shh enhances the association of Cdh7 with Gli3FL and Sufu, we speculated that Shh might alter Cdh7 distribution within the cell membrane. To address this question, we performed immunocytochemical analysis of NIH3T3 cells transiently overexpressing Cdh7. In the absence of exogenous rN-Shh, Cdh7 was broadly distributed across the cell membrane, but treatments with 2–4 nM rN-Shh caused dose-dependent Cdh7 aggregation on the cell surface (figure 5*a–d*). To quantify Shh-dependent changes in Cdh7 localization, we compared the distribution of fluorescence intensities in representative images of control-treated and rN-Shh-treated Cdh7-expressing cells by charting the number of pixels falling within specific intensity ranges (figure 5*b,c*). Most pixels in control samples fell within a restricted range of intensities, indicating that Cdh7 tends to distribute evenly across the cell surface in the absence of Shh. By contrast, rN-Shh-treated samples showed a much broader intensity distribution. This is consistent with Cdh7 aggregation resulting in Cdh7-low and Cdh7-high areas at the cell surface. Comparison of the standard deviations of fluorescence intensity in control and rN-Shh-treated samples confirmed that intensity distribution was significantly different in the absence and in the presence of rN-Shh (figure 5*d*). When the same analysis was performed with Ncdh-expressing cells, no significant differences could be detected between control and rN-Shh-treated samples, confirming that Ncdh localization at the cell surface is insensitive to Shh treatments (figure 5*e–h*). Furthermore, NIH3T3 cells transiently expressing Cdh7 showed no changes in the distribution of endogenously expressed Ncdh upon rN-Shh treatments, which promoted Cdh7 aggregation (electronic supplementary material, figure S8*a–i*).

Immunohistochemical analysis in the intermediate spinal cord region at HH st. 20 confirmed aggregation of endogenously expressed Cdh7, but not of β-catenin or Ncdh, on the surface of embryonic spinal cord cells (figure 5*i–v*). Ectopic expression of Cdh7 in the dorsal spinal cord following electroporation of a Cdh7-expressing vector resulted in significantly lower levels of Cdh7 aggregation in dorsal regions in comparison with those detected in the intermediate spinal cord region (figure 5*w–ac*), suggesting that Cdh7 aggregation is promoted in cells falling within the Shh distribution gradient.

### 2.5. Cdh7 prevents Gli3R production and promotes degradation of Gli3 protein in the presence of Shh

**Figure 6.**
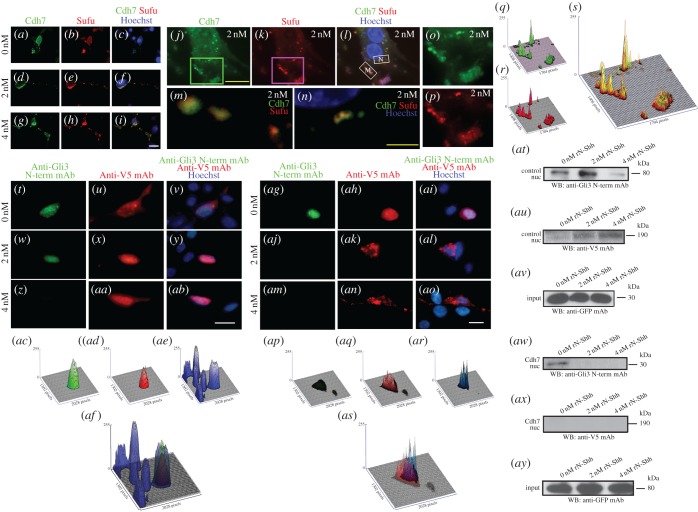
Cdh7 interacts with Sufu and collaborates with Shh to prevent Gli3R formation. (*a–s*) Immunostaining analysis with anti-Cdh7 (green signal) or anti-Sufu (red signal) antibodies using NIH3T3 cells transiently expressing Cdh7 that were cultured for 24 h in the indicated concentrations of rN-Shh. rN-Shh promotes association of Cdh7 aggregates with Sufu (*d,e,f,j,k,l*); (*m,n*) show high magnification images of the boxed areas in (*l*); (*o,p*) show high magnification images of the boxed areas in (*j,k*), respectively. Scale bar, 50 µm in (*i,j*), 10 µm in (*n*). (*q,r*) Surface plots showing quantification of fluorescence intensities in (*o,p*); (*s*) shows the merging of the plots in (*q,r*) (*t–as*). Immunostaining analysis with anti-GFP (green signal) or anti-V5 (red signal) antibodies using NIH3T3 cells transiently expressing a Gli3 chimeric construct tagged with GFP at the N-terminus and V5 at the C-terminus. Both control (*t–af*) or cells transiently expressing Cdh7 (*ag–as*) were treated for 18 h with the indicated concentrations of rN-Shh. Nuclear GFP staining is detectable in both control and Cdh7-expressing cells in the absence of rN-Shh (*t–v,ag–ah*), but not in cells treated with 4 nM rN-Shh (*z–ab,am–ao*). At 2 nM rN-Shh, nuclear GFP localization is present in control cells (*w–y*) but not in Cdh7-expressing cells (*aj–al*), suggesting that Cdh7 can effectively prevent Gli3R formation at these lower rN-Shh levels. Scale bar, 50 µm. (*ac–ae*) Surface plots of fluorescence intensities in (*w–y*); (*af*) shows the merging of the plots in (*ac–ae*). (*ap–as*) Surface plots of fluorescence intensities in (*aj, ak, al*); (*as*) shows the merging of the plots in (*aj–al*). (*at–ay*) Immunoblotting of nuclear extracts of NIH3T3 cells transiently expressing a Gli3 construct tagged with V5 at the C-terminus, using antibodies against the Gli3 N-terminal region (*at,aw*) or V5 (*au,ax*). Blots in (*at,aw*) show the 80 kDa Gli3R band, while the 190 kDa Gli3FL band is shown in (*au,ax*). Control (*at–av*) or Cdh7-expressing cells (*au–ax*) were treated with the indicated concentrations of rN-Shh. In the absence of rN-Shh, nuclear extracts from both control and Cdh7-expressing cells clearly show the 80 kDa Gli3 N-terminus-positive band, indicating that Gli3R formation takes place in these conditions. The 190 kDa V5-positive band is weakly detectable in the nuclear extract from control cells. Treatments of control cells with increasing rN-Shh levels cause progressive decrease of the ratio between the 80 kDa Gli3 N-terminus-positive and 190 kDa V5-positive bands, indicating dose-dependent inhibition of Gli3FL processing to Gli3R. In Cdh7-expressing cells, the 80 kDa band is clearly decreased and the 190 kDa band is undetectable in rN-Shh-treated samples, indicating that Cdh7 effectively prevents Gli3R production and leads to Gli3 degradation in the presence of rN-Shh. Transfection efficiency in each condition is shown by immunoblotting using anti-GFP mAb (*av,ay*), following co-transfection of a GFP control plasmid.

As Shh is able to alter Cdh7 distribution at the cell membrane, we investigated whether Cdh7 aggregates forming in the presence of Shh co-localized with Sufu. We expressed Cdh7 in NIH3T3 cells and cultured them in the presence of 0, 2 or 4 nM rN-Shh. We then incubated non-permeabilized cells with anti-Cdh7 mAb, followed by cell permeabilization and incubation with anti-Sufu polyclonal antibody (pAb) (0 nM rN-Shh, figure 6*a–c*; 2 nM rN-Shh, figure 6*d–f*, *j–s*; 4 nM rN-Shh, figure 6*g–i*). In the absence of exogenous rN-Shh, Cdh7 was present across the entire cell membrane and Sufu was weakly detectable in the cytosol (figure 6*a–c*). Following rN-Shh treatments, Cdh7 aggregates were associated with Sufu at the cytoplasmic side of the cell membrane (figure 6*d–s*), while no association was detectable between Sufu and Ncdh, Cdh20 or a truncated Cdh7 version lacking the intracellular domain (electronic supplementary material, figure S9 and figure S10*a–i*). Artificially cross-linking Cdh7 molecules on the cell surface using anti-Cdh7 mAb resulted in association with Sufu, but could not activate GBS-luc reporter expression, indicating that Cdh7 aggregation *per se* is not sufficient to promote Shh signalling (electronic supplementary material, figure S10*j–m*). These results suggest that Cdh7 positively modulates Shh signalling by interacting with Sufu at the intracellular level and that this interaction involves Shh-dependent regulation of Cdh7 distribution within the cell membrane.

To investigate whether Cdh7 interaction with Gli3FL and Sufu can modulate Gli3R production, we generated a construct encoding for a fusion protein of mouse Gli3FL with an N-terminal GFP tag and a C-terminal V5 tag (GFP-*Gli3FL*-V5). This allowed to detect nuclear accumulation of Gli3R as positive GFP nuclear staining together with negative V5 staining, while co-localization of GFP and V5 staining or the absence of GFP staining would indicate decreased production of Gli3R. We transfected GFP-*Gli3FL*-V5 along with either control or Cdh7-expressing vectors into NIH3T3 cells, followed by cell culture in the presence of 0, 2 or 4 nM rN-Shh and immunocytochemical localization of GFP-tagged and/or V5-tagged peptides.

In control untreated cells, we detected nuclear GFP staining, while V5 staining was mainly localized in the cytoplasm, indicating efficient production and nuclear localization of Gli3R (figure 6*t–v*). Treatments with 2 nM rN-Shh resulted in nuclear co-localization of both GFP and V5 staining, suggesting that in the presence of Shh signalling Gli3 protein was less efficiently converted into Gli3R (figure 6*w–y,ac–af*). No GFP staining was detectable after treatments with 4 nM rN-Shh (figure 6*z–ab*), indicating that elevated levels of Shh signalling can prevent Gli3R formation. Nuclear V5 staining was detectable at these doses of rN-Shh, suggesting that they cause Gli3 protein degradation to C-terminal fragments [12], which translocate into the nucleus. These degradation products are likely to coexist with Gli3R at lower rN-Shh doses (2 nM), while they predominate over Gli3R at higher rN-Shh doses (4 nM). In the absence of rN-Shh, Cdh7 expression had no impact on Gli3R formation (figure 6*ag–ai*). No GFP staining, however, was detectable after treatments of Cdh7-expressing cells with 2 nM rN-Shh (figure 6*aj,ap–as*), suggesting that Cdh7 efficiently blocks Gli3R production in the presence of these lower doses of rN-Shh. V5 staining was largely excluded from the nucleus in Cdh7-expressing cells treated with rN-Shh (figure 6*ak,al,an,ao,ap–as*), suggesting that C-terminal fragments resulting from Gli3 degradation in the presence of both rN-Shh and Cdh7 do not undergo nuclear translocation. V5 staining and Cdh7 aggregate staining, however, did not co-localize (electronic supplementary material, figure S11), indicating that Gli3 C-terminal fragments forming in these conditions do not associate with Cdh7 aggregates.

Immunoblotting analysis using nuclear extracts led to results consistent with immunostaining data. In this case, cells were transfected with a construct encoding for Gli3FL-V5 fusion protein, with or without Cdh7-expressing vector, and an antibody against the Gli3 N-terminal region was used to detect Gli3R or Gli3FL. Cells were co-transfected with a control GFP vector to account for transfection efficiency. In agreement with immunostaining results, a nuclear 80 kDa Gli3R band was detectable with anti-Gli3 N-terminus antibody both in the absence of rN-Shh and in the presence of 2 nM rN-Shh, but it was reduced to approximately half of the levels detected in untreated cells after treatments with 4 nM rN-Shh (figure 6*at,aw*; western blot quantifications are reported in electronic supplementary material, table S6). By contrast, the levels of nuclear 190 kDa Gli3FL band, as detected with anti-V5 antibody, were roughly doubled in rN-Shh-treated cells in comparison with untreated cells (figure 6*au,av*; electronic supplementary material, table S6). These results confirm the idea that Gli3R formation can take place at lower doses of rN-Shh (2 nM) but it is efficiently inhibited at higher rN-Shh doses (4 nM). They further suggest that rN-Shh treatments lead to Gli3 protein degradation to C-terminal fragments along with limited nuclear accumulation of Gli3FL (which is detectable by immunoblotting, but not by immunostaining). Notably, in Cdh7-expressing cells treated with 2 nM rN-Shh, the nuclear Gli3R band was decreased to levels comparable to those present in cells treated with 4 nM rN-Shh and roughly corresponding to half of the levels detected in the absence of rN-Shh (figure 6*aw, ay*; electronic supplementary material, table S6). These results confirm that Cdh7 can inhibit Gli3R formation in the presence of low/moderate doses of rN-Shh. No V5-positive 190 kDa Gli3FL band was detectable by immunoblotting in nuclear extracts of Cdh7-expressing cells (figure 6*ax, ay*; electronic supplementary material, table S6), indicating that, in the presence of rN-Shh, Cdh7 causes efficient Gli3 protein degradation to Gli3 C-terminal fragments.

We confirmed that, in immunostaining assays, detection of V5 signal without GFP signal was due to C-terminal Gli3 peptides forming as a result of Gli3 degradation by means of immunoblotting assays with cytoplasmic extracts of cells transfected with *Gli3FL*-V5 using antibodies against the Gli3 N-terminal region or V5 tag. While no 80 kDa Gli3R or 190 kDa Gli3FL bands were detectable, we observed several low molecular weight bands of various sizes (20–70 kDa) by immunoblotting with anti-V5 antibodies (electronic supplementary material, figure S12). This suggests that Gli3FL is unstable in the cytoplasm and undergoes partial proteolysis generating C-terminal fragments. Co-transfection of plasmids expressing Cdh7 and siRNA-*Cdh7* prevented the effects of Cdh7 on both Sufu localization (electronic supplementary material, figure S13*a–i*) and Gli3 processing (electronic supplementary material, figure S13*j–r*), indicating their specificity.

Taken together, these results indicate that, by associating with Sufu and Gli3FL, Cdh7 can effectively collaborate with Shh to prevent formation of Gli3R, leading to Gli3FL cytoplasmic degradation and enhanced activation of Shh signalling.

## 3. Discussion

A gradient of Shh protein, released from the notochord and floor plate, plays a crucial role in ventral neural tube patterning [2,5,6,29]. Analysis of a variety of mutant mice, lacking different components of the Shh signalling pathway, shows that Shh-dependent patterning extends up to a sharp boundary between Pax7-negative and Pax7-positive domains in the ventral and dorsal neural tube, respectively [2,5,6,29]. As a result of Shh graded action, the Pax7^−^ region becomes subdivided into the floor plate and five sharply delimited neural progenitor domains (p3, pMN, p2–p0), each of them expressing specific transcription factors [2,5,6,29].

A crucial question that remains only partially addressed is how an apparently continuous gradient of Shh protein results in distinct, well-defined boundaries of gene expression at different levels of the spinal cord DV axis. A crucial element in the interpretation of the Shh gradient appears to be the regulatory architecture of the transcriptional network activated by Shh signalling, which involves cross-repressive interactions between different transcription factors [30–32]. This mechanism ensures both activation of distinct genes at different thresholds of Shh signalling, and the establishment of mutually exclusive gene expression domains delimited by sharp borders [30–32]. Although much progress has been made to clarify how floor plate, p3, pMN and p2 domains are specified in the ventral spinal cord, the molecular mechanisms establishing the boundary between the Pax7^−^ and Pax7^+^ regions in the intermediate spinal cord remain poorly investigated.

**Figure 7.**
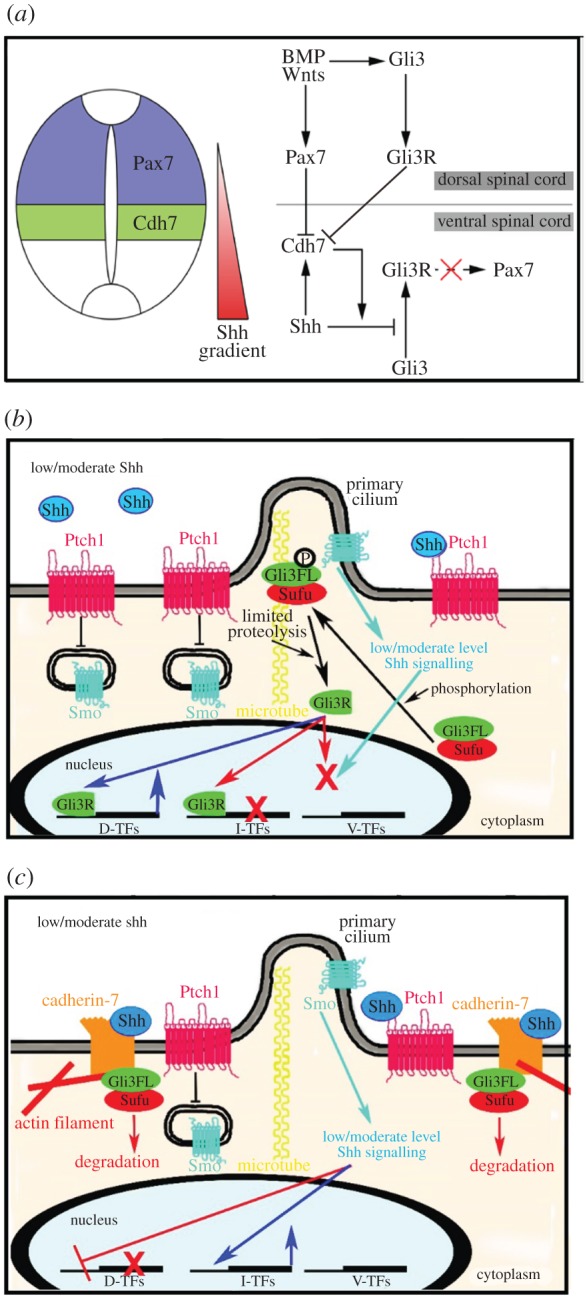
Models of Cdh7 roles in the regulation of Shh signalling and neural tube patterning. (*a*) Model of Cdh7-dependent specification of the Pax7^+^/Pax7^−^ neural tube boundary via regulation of Gli3R production and Pax7 expression. Shh promotes Cdh7 expression in the intermediate spinal cord region and collaborates with Cdh7 to prevent processing of Gli3 to Gli3R and Pax7 expression. Dorsal to the Cdh7 expression domain, Gli3 is converted into Gli3R, indirectly leading to Pax7 expression (dashed arrow). Repression of Cdh7 expression by Pax7 helps to maintain a sharp boundary between the Cdh7^+^/Pax7^−^ ventral spinal cord and the Cdh7^−^/Pax7^+^ dorsal spinal cord. BMPs and Wnts promote both Gli3 and Pax7 expression in the dorsal spinal cord. (*b,c*) Models of the regulation of Gli-dependent signalling in cells exposed to low/moderate levels of Shh in the absence (dorsal spinal cord cell, (*b*)) or in the presence (ventral spinal cord cell, (*c*)) of Cdh7. In the dorsal spinal cord (Cdh7 non-expressing cell), low/moderate Shh levels do not effectively prevent the Gli3FL/Sufu complex from transiting through the base of the primary cilium, where Gli3FL becomes partially phosphorylated and converted into Gli3R, leading to expression of dorsal transcription factors (D-TFs) and repression of intermediate and ventral neural tube TFs (I-TFs, V-TFs) (*b*). In ventral spinal cord cells (Cdh expressing cell), low/moderate Shh levels collaborate with Cdh7 to retain the Gli3FL/Sufu complex in the cytoplasm, thus preventing Gli3R formation and leading to Gli3 degradation and expression of I-TFs (*c*). Higher Shh levels are needed for expression of V-TFs (not shown). See text for further details.

Cdh7 is expressed on the ventral side of the Pax7^+^/Pax7^−^ boundary in the intermediate spinal cord region overlapping with the dorsal tail of the Shh gradient, and its dorsal limit of expression is confined to the ventral border of the Pax7^+^ region (figure 7*a*). Confirming previous results [18], we found that Cdh7 expression in this intermediate spinal cord domain is dependent on low/moderate Shh levels. We show for the first time that Cdh7 overexpression represses Pax7 expression, while Cdh7 knock-down causes ventral expansion of Pax7^+^ domain. In these assays, Cdh7 could inhibit Pax7 expression only in the proximity of the Pax7^+^/Pax7^−^ boundary, but not in the most dorsal spinal cord region, which is devoid of Shh. Therefore, Shh promotes Cdh7 expression in the intermediate spinal cord and also collaborates with Cdh7 to negatively regulate Pax7 expression. Supporting this idea, we found that Cdh7 interacts with Shh protein and cooperates with Shh to prevent Gli3R formation. This interaction boosts Shh signalling at low/moderate doses of the Shh ligand. We speculate that this Cdh7-dependent mechanism of Shh pathway enhancement leads to a sharp drop in the levels of Shh signalling on the dorsal side of the Cdh7^−^/Cdh7^+^ boundary with respect to cells on the ventral side of the border. This, in turn, facilitates establishment of Pax7^−^ and Pax7^+^ domains on each side of this border.

Based on these observations, we propose the model shown in figure 7*a*. According to this model, ventral to the Cdh7^−^/Cdh7^+^ boundary, Shh-dependent Cdh7 expression reinforces Shh signalling by inhibiting formation of Gli3R, thus preventing Pax7 expression and specification of dorsal identities. Dorsal to the Cdh7^−^/Cdh7^+^ boundary, however, lower levels of Shh in the absence of Cdh7 are not sufficient to prevent Gli3R formation and Pax7 expression. As previously described [33–36], BMP and Wnt signals play a major role in promoting Pax7 expression in the dorsal spinal cord either directly or indirectly via positive regulation of Gli3 expression, while Pax7-dependent repression of Cdh7 expression [18,37] probably contributes to sharpening the boundary between the Cdh7^+^/Pax7^−^ and Cdh7^−^/Pax7^+^ regions. One possible caveat to this model comes from previous reports showing that the Pax7^+^ domain in the dorsal spinal cord is not affected in Gli3 mutant mice [38], suggesting that Cdh7 may also modulate Pax7 expression via Gli3-independent mechanisms. It should be noted, however, that the Pax7^+^ domain spreads ventrally in either Shh or Smo mutant mice, whereas it is correctly confined to the dorsal spinal cord in Shh/Gli3 or Smo/Gli3 double mutant mice [38–40]. Therefore, inhibition of Gli3R formation in the intermediate spinal cord is both necessary and sufficient to ventrally delimit the Pax7^+^ domain, even though Pax7 expression in the dorsal spinal cord can be maintained independently of Gli3. Although Cdh7-dependent homophilic interactions may also have a role in refining the Pax7^+^/Pax7^−^ boundary and delimiting coherent progenitor domains, Cdh7-dependent regulation of Shh signalling clearly plays a major role in this process, because Cdh7 overexpression in the dorsal neural tube, outside of the Shh range, was unable to affect Pax7 expression and alter DV patterning. Further work will be needed to elucidate possible roles of Gli3-independent and/or cell adhesion-dependent mechanisms of Cdh7 function in spinal cord patterning.

In spite of continuous progress in the field of Shh signal transduction, the biochemical machinery controlling processing of Gli proteins in the absence or in the presence of Shh remains only partially understood. According to recent interpretations [10,41,42], in the absence of Shh, Gli2/3 proteins bind Sufu in the cytoplasm and transit through the primary cilium. When crossing the base of the primary cilium, Gli2/3FL undergo phosphorylation by PKA, GSK3β and CKI, leading to their conversion into Gli2/3R. While Gli2R is degraded, Gli3R accumulates in the nucleus and represses transcription of Shh-target genes. Activation of Shh signalling is thought to increase the residence time of Gli2/3FL–Sufu complexes within the primary cilium, thus protecting Gli2/3FL from PKA/GSK3β/CKI-dependent phosphorylation and preventing formation of Gli2/3R. This leads to nuclear accumulation of Gli2FL, causing activation of Shh targets, while unphosphorylated GLI3FL appears to be quickly degraded by the proteasome [10–12,41].

We found that the extracellular region of Cdh7 binds Shh and this interaction promotes the association of Cdh7 with Sufu on the intracellular side of the cell membrane. We also found that Shh stimulates Cdh7 aggregation, suggesting that Cdh7 movement within the cell membrane is reduced in the presence of Shh and that a mechanism involving Shh-dependent Cdh7 translocation to the primary cilium is unlikely. Preliminary evidences suggest that Cdh7 is excluded from the primary cilium either in the presence or in the absence of Shh (data not shown). Based on these observations, we propose that, following binding to Shh, Cdh7 associates with Sufu and retains the Sufu–Gli3FL complex in the cytoplasm, thus preventing its translocation at the base of the primary cilium and Gli3FL processing to Gli3R (figure 7*b,c*). As we observed extensive degradation of the Gli3 protein in the presence of both Cdh7 and Shh, we speculate that inhibiting Sufu–Gli3FL movement to the base of the primary cilium and Gli3FL phosphorylation by PKA/GSK3β/CKI eventually leads to Gli3FL dissociation from Sufu and degradation, irrespective of whether this involves increased residence of the Sufu–Gli3FL complex within the primary cilium (in the presence of high Shh levels without Cdh7) or within the cytoplasm (in the presence of both Cdh7 and low/moderate Shh levels). It should be noted, however, that Cdh7-dependent activation of Shh signalling is sensitive to Smo inhibitors and is enhanced by an active form of Smo, indicating that Smo-mediated signal transduction, possibly involving the primary cilium, is required for Shh signalling activation even in the presence of Cdh7. Cdh7 ability to enhance Shh signalling was limited to low/moderate doses of Shh. No Cdh7-dependent increase in Shh signalling was detectable at higher Shh levels, suggesting that the cross-talk between Cdh7 and Shh is likely to involve complex, dose-dependent mechanisms. Future investigations will need to shed light on how Cdh7-dependent and Smo-dependent mechanisms converge on the regulation of Shh-target genes, and on how the interactions between Cdh7 and the Shh signalling pathway are influenced by the levels of the Shh ligand.

In conclusion, Cdh7 expressed in the intermediate region of the vertebrate spinal cord is required for proper neural tube patterning. This role is due to the ability of Cdh7 to enhance Shh signalling in the presence of low/moderate levels of Shh, by causing more efficient inhibition of Gli3R formation than would be achieved by these levels of Shh on their own. We propose that this mechanism leads to a marked difference in the levels of Shh signalling between Cdh7^+^ and Cdh7^−^ cells exposed to limited amounts of the Shh ligand. This, along with opposite regulation of Cdh7 expression by Shh and Pax7, ensures establishment of a sharp boundary between the Pax7^+^ and Pax7^−^ neural tube regions. By focusing mainly on the role of transcription factors, previous landmark studies have shown that the interpretation of the Shh gradient depends on the regulatory logic of downstream molecular networks [30–32,43], and this work excitingly shows how embedding transmembrane proteins such as Cdh7 in Shh-dependent molecular networks can set the stage for morphogen-dependent tissue patterning.

## 4. Material and methods

Detailed description of experimental procedures is available in the electronic supplementary material.

## Supplementary Material

Supplementary Figures; Supplementary Tables; Supplementary Materials and Methods

## Data accessibility

Additional data are available in the electronic supplementary material.
