# Supplementary material for: Cadherin-7 enhances Sonic Hedgehog signalling by preventing Gli3 repressor formation during neural tube patterning
Source: Open Biol. 2017 Dec 20;7(12):170225. doi: 10.1098/rsob.170225 (PMC5746549; doi:10.1098/rsob.170225)
Supplement: Supplementary Figures; Supplementary Tables; Supplementary Materials and Methods [file rsob170225supp1.pdf]

Supplementary Figures

Figure S1

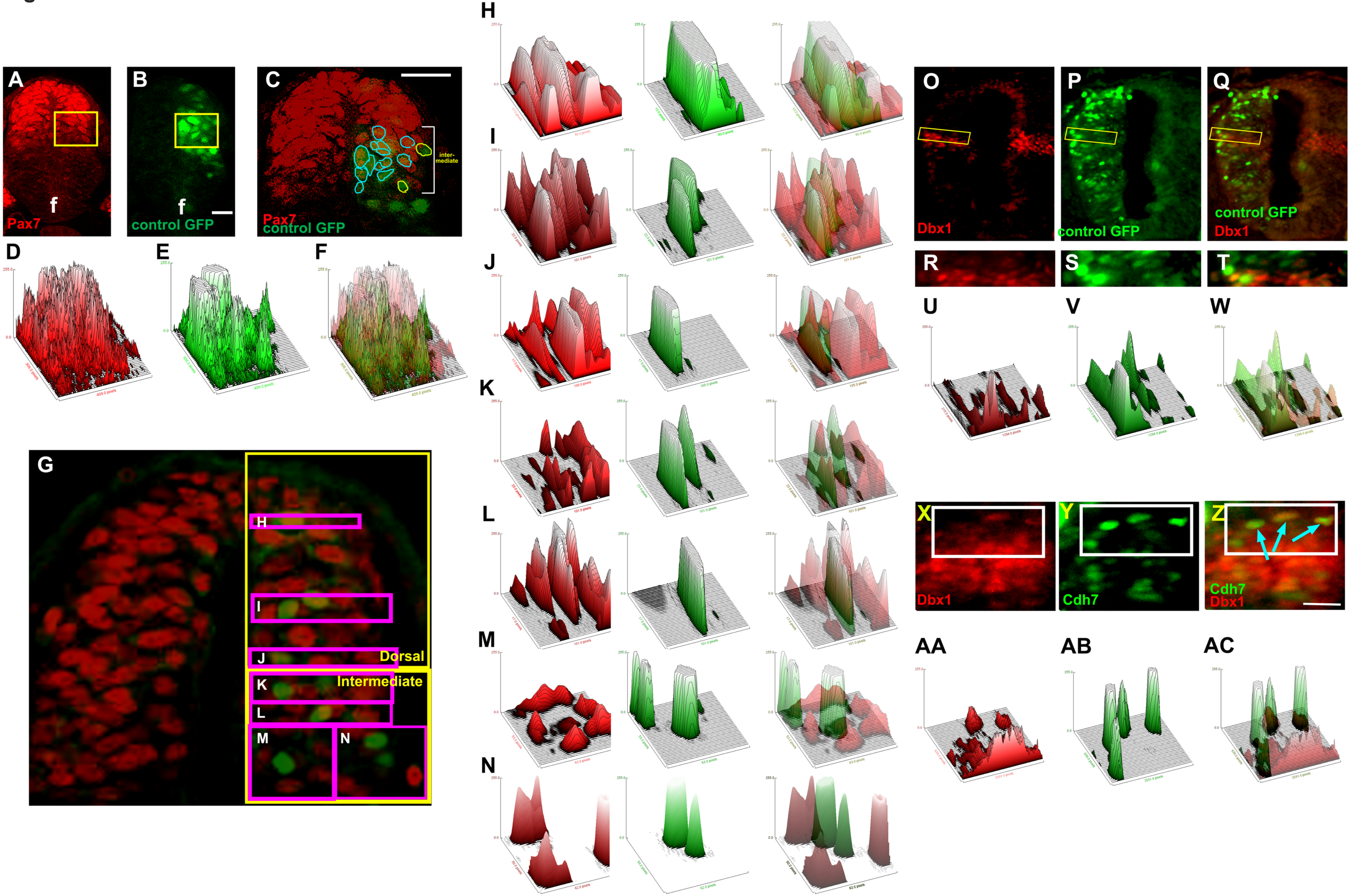

A
B
C
inter-
mediate
f
f
Pax7
control GFP
Pax7
control GFP
D
E
F
G
H
I
J
Dorsal
K
Intermediate
L
M
N
O
P
Q
Dbx1
control GFP
control GFP
Dbx1
R
S
T
U
V
W
X
Y
Z
Dbx1
Cdh7
Cdh7
Dbx1
AA
AB
AC

Figure S1. Cdh7 inhibits Pax7 and promotes Dbx1 expression in the intermediate region of the chick embryonic spinal cord (related to Figure 2).

(A-C) Immunostaining with anti-Pax7 (red staining) and anti-GFP (green staining) antibodies on HH st.15 neural tube, following unilateral electroporation at HH st. 10 of a control plasmid driving expression of GFP protein fused to a nuclear localization signal. **(D-F)** Surface plot quantification of fluorescent intensities in the squared or bracketed areas in **(A-C)**. GFP positive cells are nearly all Pax7-positive.

(G) Immunostaining with anti-Pax7 (red staining) and anti-GFP (green staining) antibodies on HH st.15 neural tube, following unilateral electroporation at HH st. 10 of a control plasmid driving expression of both Cdh7 and a chimeric GFP protein fused to a nuclear localization signal. **(H-N)** Surface plot quantification of fluorescent intensities in the squared areas in **(G)**. Cdh7-expressing cells (GFP-positive) are Pax7-positive in the dorsal spinal cord **(H-J)** and mostly Pax7-negative in the intermediate neural tube region **(K-N)**.

(O-W) Immunostaining analysis with anti-Dbx1 (red staining) and anti-GFP (green staining) antibodies on HH st.15 neural tube, following unilateral electroporation at HH st. 10 of a control plasmid driving expression of GFP protein fused to a nuclear localization signal. **(R-T)** show higher magnification of the boxed regions in **(O-Q)**. Surface plot quantification of fluorescent intensities in **(R-T)** is shown in **(U-W)**. GFP positive cells dorsal to Dbx1 endogenous domain are Dbx1-negative.**(X-AC)** Immunostaining with anti-Dbx1 (red staining) and anti-GFP (green staining) antibodies on HH st.15 neural tube, following unilateral electroporation at HH st. 10 of a control plasmid driving expression of both Cdh7 and a chimeric GFP protein fused to a nuclear localization signal. Surface plot quantification of fluorescent intensities in **(X-Z)** is shown in **(AA-AC)**. GFP positive cells dorsal to Dbx1 endogenous domain ectopically express Dbx1.

Figure S2

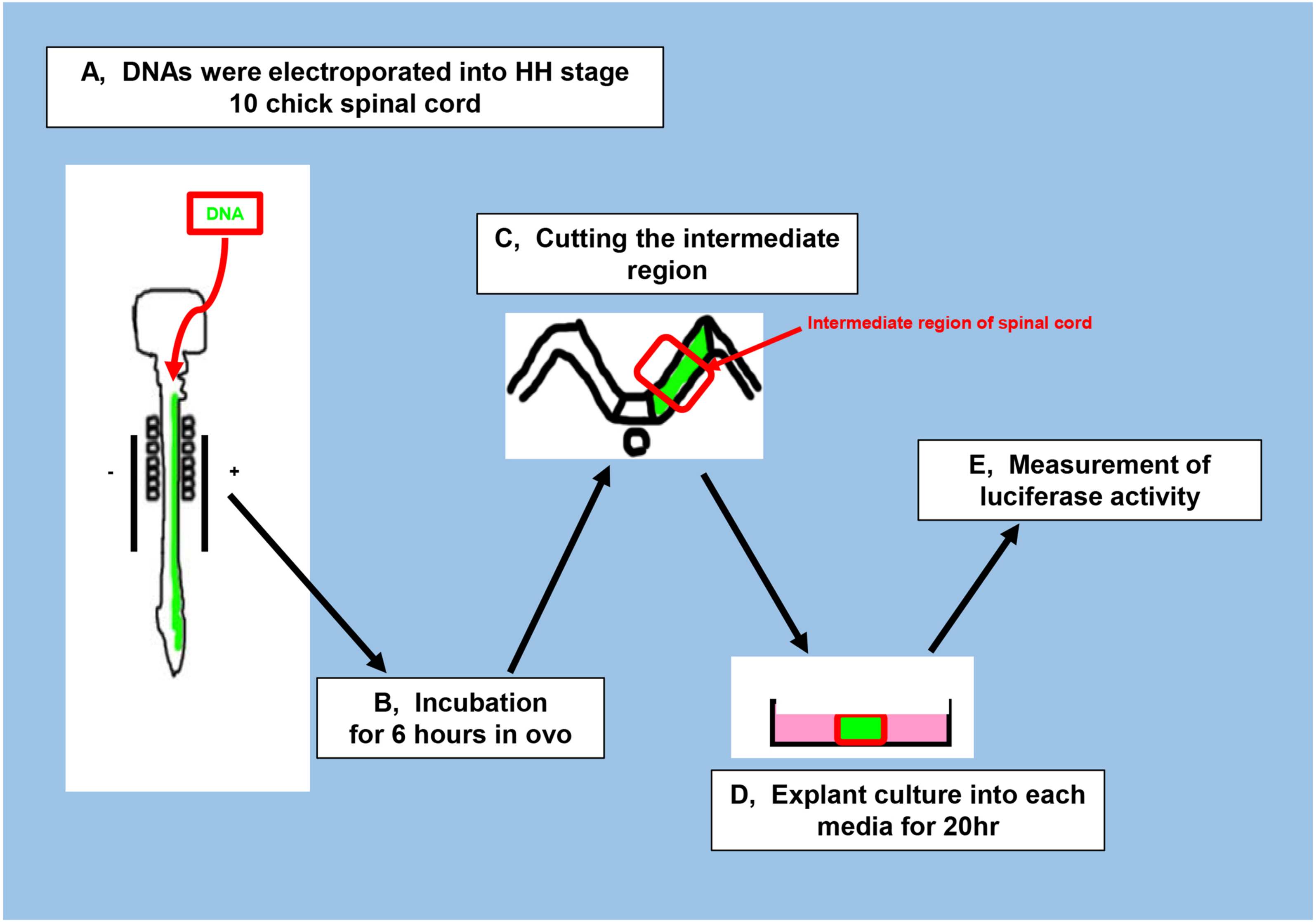

Modified method of Dessaud, E. et al. (2007)

Figure S2. Schematic diagram of the experimental procedure for GBS-luc reporter assays in chick spinal cord explants (related to Figure 3).

HH st. 10 chick embryos were electroporated *in ovo* with a plasmid mixture including the desired expression plasmids, GBS-luc reporter plasmid and an internal control plasmid. After 6 hours, explants of presumptive intermediate spinal cord were dissected, cultured *in vitro* for 20 hours in control media or in media containing rN-Shh and/or 25nM cyclopamine-KAAD and assayed for RLA.

Figure S3

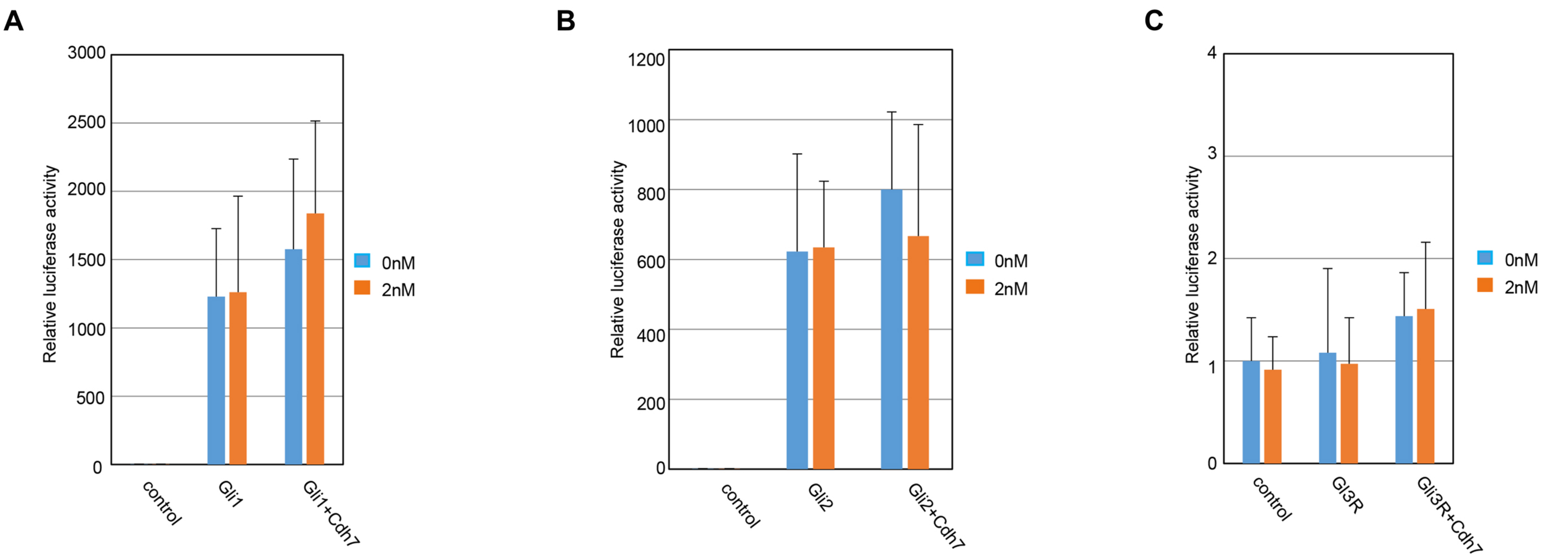
A
3000
2500
2000
1500
1000
500
0
Relative luciferase activity
control
Gli1
Gli1+Cdh7
0nM
2nM
B
1200
1000
800
600
400
200
0
Relative luciferase activity
control
Gli2
Gli2+Cdh7
0nM
2nM
C
4
3
2
1
0
Relative luciferase activity
control
Gli3R
Gli3R+Cdh7
0nM
2nM

Figure S3. Cdh7 does not regulate Shh signaling through Gli1, Gli2, or Gli3R (related to Figure 3).

(A-C) RLA quantification in HH st.10 explants of presumptive intermediate spinal cord, which were dissected from embryos electroporated with GFP (control) or the indicated expression plasmids along with GBS-luc reporter and internal control plasmids, and treated with the indicated doses of rN-Shh as described in the supplementary Materials and Methods. Cdh7 cannot enhance Shh signaling in combination with both Gli1 **(A)**, Gli2 **(B)** or Gli3R **(C)** and 2 nM rN-Shh. Results shown represent the mean ± SEM of at least 10 experiments.

Figure S4

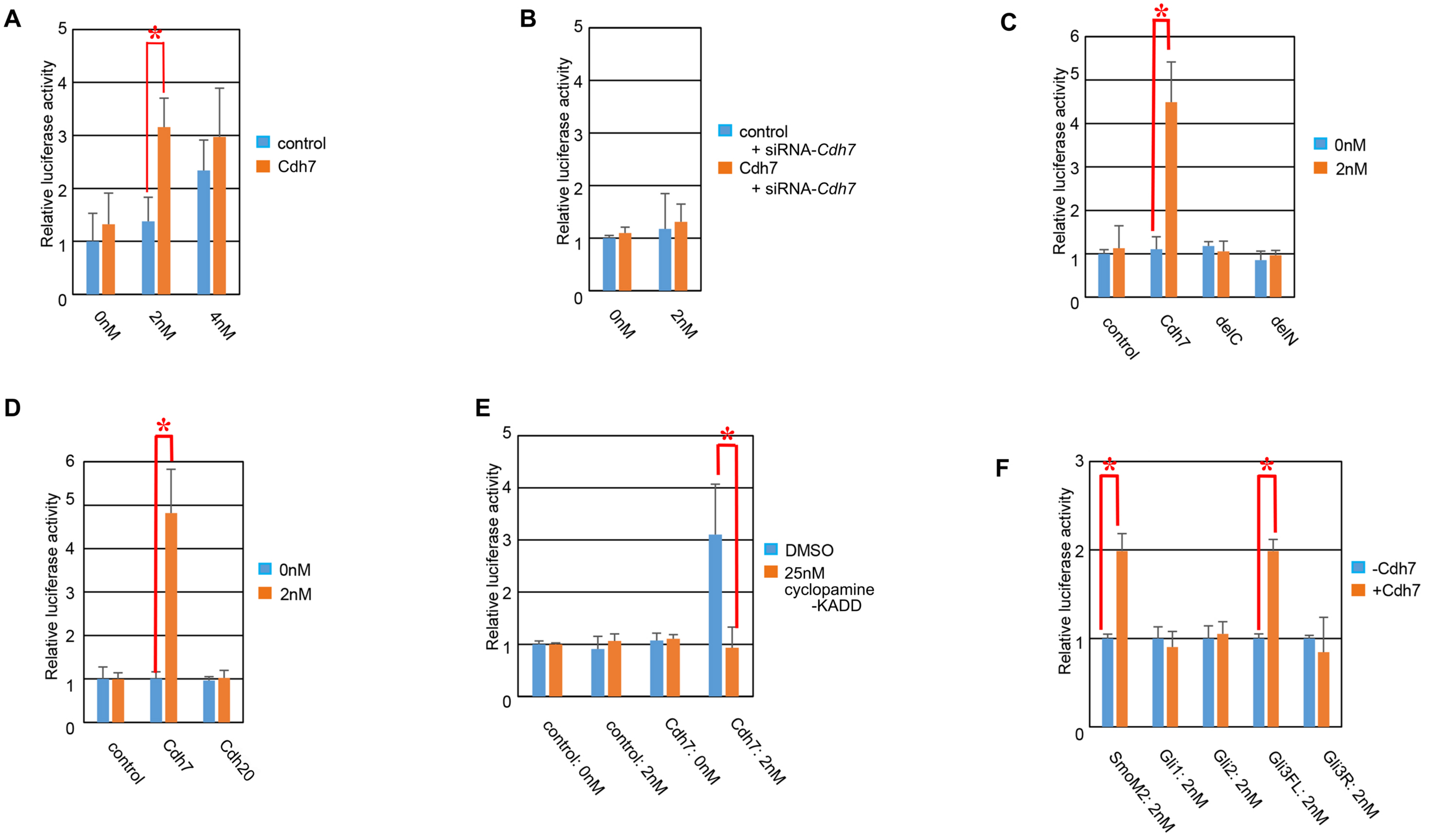

A
Relative luciferase activity
0
1
2
3
4
5
0nM
2nM
4nM
control
Cdh7
*
B
Relative luciferase activity
0
1
2
3
4
5
0nM
2nM
control
+ siRNA-*Cdh7*
Cdh7
+ siRNA-*Cdh7*
C
Relative luciferase activity
0
1
2
3
4
5
6
control
Cdh7
delC
delN
0nM
2nM
*
D
Relative luciferase activity
0
1
2
3
4
5
6
control
Cdh7
Cdh20
0nM
2nM
*
E
Relative luciferase activity
0
1
2
3
4
5
control: 0nM
control: 2nM
Cdh7: 0nM
Cdh7: 2nM
DMSO
25nM
cyclopamine
-KADD
*
F
Relative luciferase activity
0
1
2
3
SmoM2: 2nM
Gli1: 2nM
Gli2: 2nM
Gli3FL: 2nM
Gli3R: 2nM
-Cdh7
+Cdh7
*
*

Figure S4. Cdh7 enhances Shh signaling by acting at the level of Gli3FL in NIH3T3 cells (related to Figure 3).

(A) RLA quantification in NIH3T3 cells that were transfected with pCAGGS vector (control) or Cdh7 expression plasmids along with GBS-luc reporter and internal control plasmids, and treated for 20 hours with the indicated doses of rN-Shh as described in the supplementary Materials and Methods. Cdh7 overexpression significantly enhances RLA in the presence of 2 nM rN-Shh, but not in untreated explants or in the presence of 4 nM rN-Shh. Results are shown as the mean of at least 6 independent experiments. Error bars represent SEM. *, $p<0.005$ according to Student's t-test.

(B) RLA quantification in cells that were treated as in **(A)** but also transfected with Cdh7-targeting siRNA plasmid (siRNA-*Cdh7*), confirming that Cdh7 knock-down abolishes the effects of Cdh7 overexpression.

(C) RLA quantification in cells that were treated as in **(A)** but transfected with Cdh7 deletion constructs in place of full-length Cdh7, showing that the ability of Cdh7 to enhance Shh signaling requires both Cdh7 extra-cellular and intra-cellular regions.

(D) RLA quantification in cells that were treated as in **(A)** but electroporated with a plasmid encoding for Cdh20 instead of Cdh7,

showing that the ability of Cdh7 to enhance Shh signaling is not shared by Cdh20.

(E) RLA quantification in cells that were treated as in **(A)** but also exposed to DMSO or 25nM cyclopamine-KAAD, showing that Smo inhibition abrogates the ability of Cdh7 to enhance Shh signaling.

(**F)** RLA quantification in cells that were treated as in **(A)** but transfected with either SmoM2, Gli1, Gli2, Gli3FL or Gli3R expression constructs with or without Cdh7 encoding plasmid, showing that Cdh7 can further enhance Shh signaling in combination with 2 nM rN-Shh and either SmoM2 or Gli3FL, but not Gli1, Gli2 or Gli3R.

Figure S5

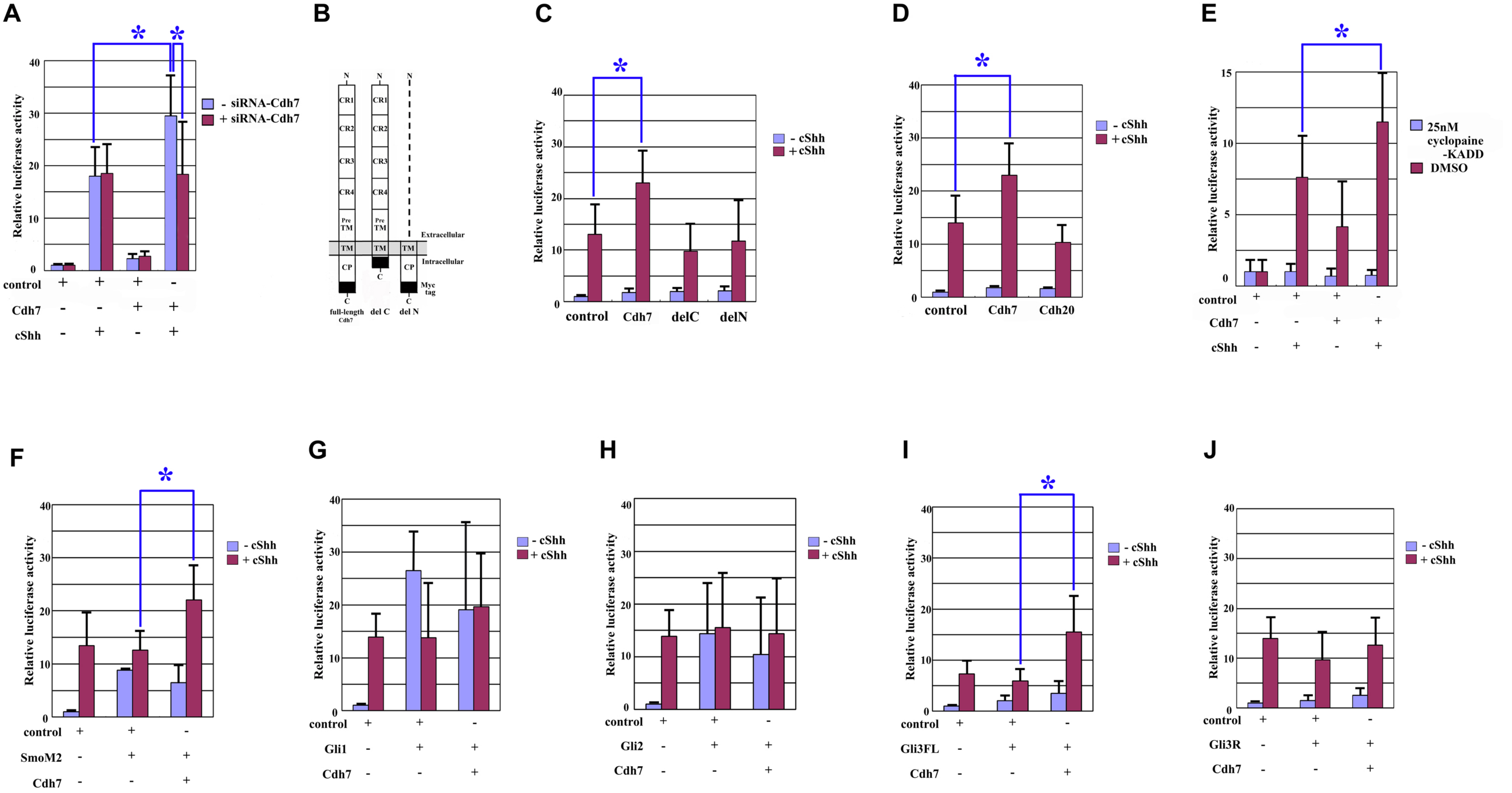

A
B
C
D
E
F
G
H
I
J
Relative luciferase activity
- siRNA-Cdh7
+ siRNA-Cdh7
control
Cdh7
cShh
N
CR1
CR2
CR3
CR4
Pre TM
TM
CP
C
Extracellular
Intracellular
Myc tag
full-length Cdh7
del C
del N
- cShh
+ cShh
delC
delN
Cdh20
25nM cyclopaine -KADD
DMSO
SmoM2
Gli1
Gli2
Gli3FL
Gli3R

Figure S5. Cdh7 can enhance Shh signaling in combination with cShh-expressing plasmid (related to Figure 3).

(A) RLA quantification in HH st.10 explants of presumptive intermediate spinal cord, which were dissected from embryos electroporated with different combinations of Cdh7, cShh or siRNA-*Cdh7* expression plasmids along with GBS-luc reporter and internal control plasmids, as indicated and as described in the supplementary Materials and Methods. Cdh7 overexpression significantly enhances RLA in the presence, but not in the absence, of cShh plasmid, and this effect is abrogated by siRNA-*Cdh7* plasmid. Results are shown as the mean of 12 independent experiments. Error bars represent SEM. *, $p<0.005$ according to Student's t-test.

(B) Schematic diagram of myc-tagged Cdh7 full-length and deletion constructs as used for the assays shown in **(C)**.

(C) RLA quantification in explants that were treated as in **(A)**, but electroporated with Cdh7 deletion constructs in place of full-length Cdh7. Cdh7 requires both its extra-cellular and intra-cellular regions to enhance Shh signaling.

(D) RLA quantification in explants that were treated as in **(A)**, but electroporated with constructs encoding for Cdh20 instead of

Cdh7. Cdh7 ability to enhance Shh signaling is not shared by Cdh20.

(E) RLA quantification in explants that were treated as in **(A)**, but also exposed to DMSO or 25nM cyclopamine-KAAD as indicated. Smo inhibition abrogates the ability of Cdh7 to enhance Shh signaling.

(F) RLA quantification in explants that were treated as in **(A)**, but also electroporated with SmoM2 expression construct, as indicated. Cdh7 can further enhance Shh signaling in the presence of both SmoM2 and cShh.

(G-J) RLA quantification in explants that were treated as in **(A)**, but also electroporated with either Gli1 **(G)**, Gli2 **(H)**, Gli3FL **(I)** or Gli3R **(J)** expression constructs, as indicated. Cdh7 can enhance Shh signaling in combination with both cShh and Gli3FL, but not Gli1, Gli2 and Gli3R.

Figure S6

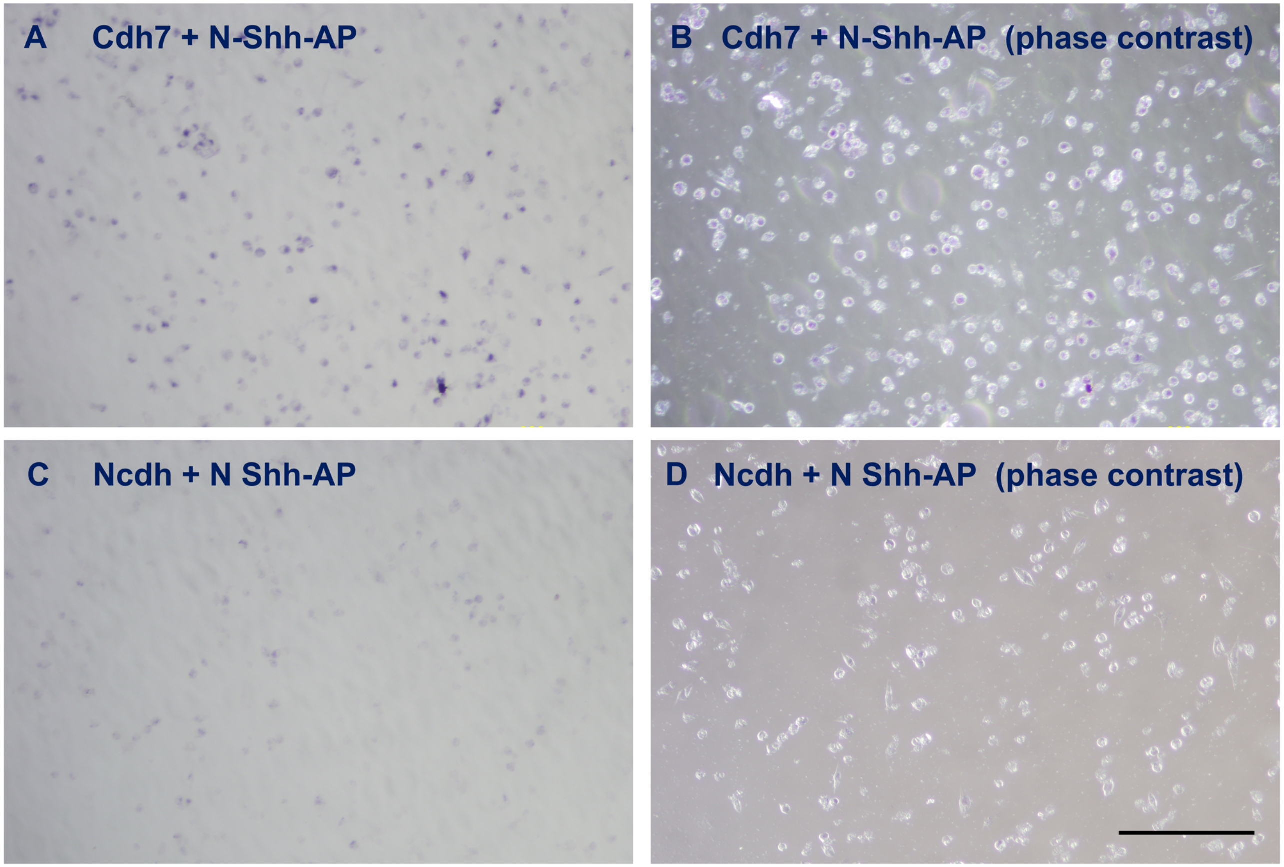

A Cdh7 + N-Shh-AP
B Cdh7 + N-Shh-AP (phase contrast)
C Ncdh + N Shh-AP
D Ncdh + N Shh-AP (phase contrast)

Figure S6. N-Shh interacts with Cdh7 but not Ncdh (related to Figure 4).

(A-D) AP staining of Chd7-expressing **(A, B)** or Ncdh-expressing **(C, D)** COS-7 cells that were incubated with conditioned medium from 293 cells expressing N-Shh-AP. N-Shh-AP signal is detectable in Cdh7-expressing cells, but not in Ncdh-expressing cells. **(B)** and **(D)** are phase-contrast images of the fields shown in **(A)** and **(C)**. Scale bar, 100 µm.

Figure S7

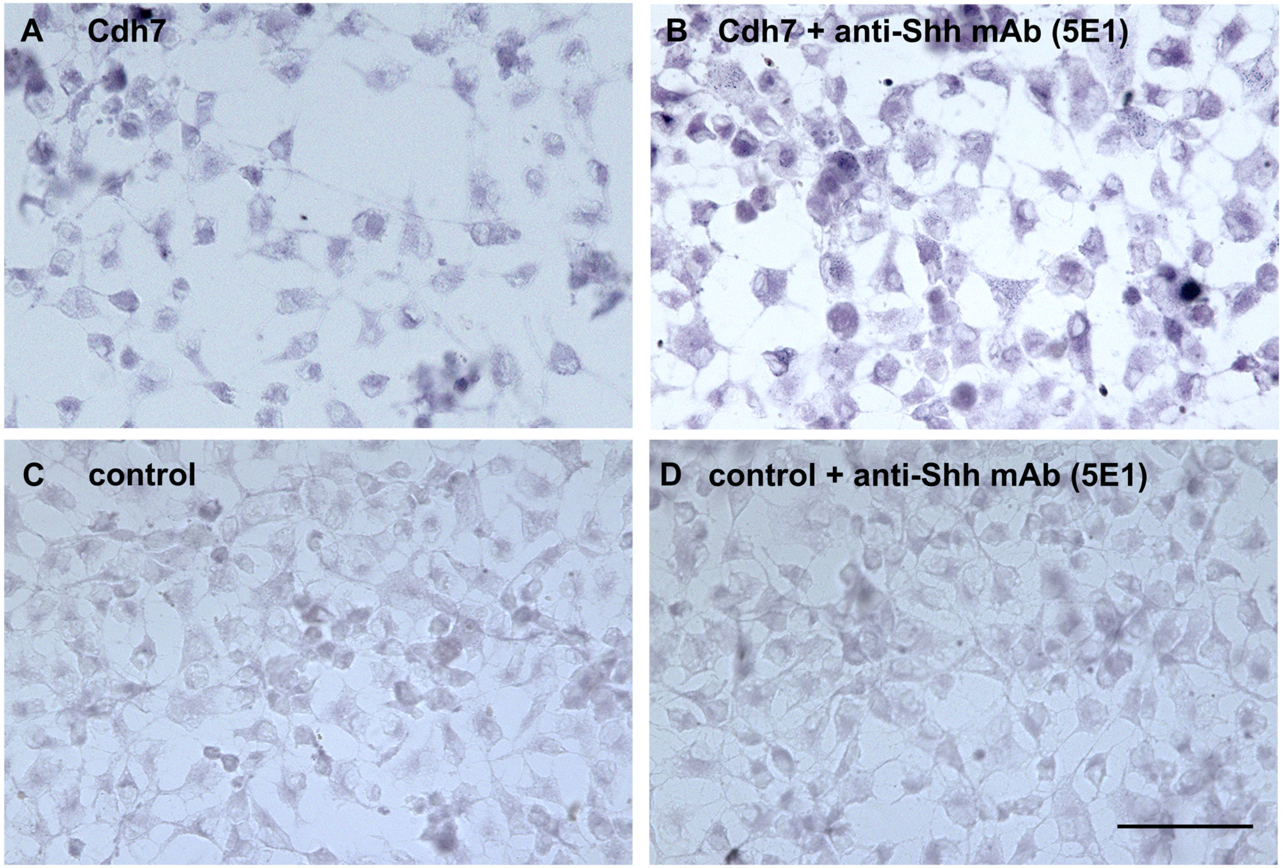
A Cdh7
B Cdh7 + anti-Shh mAb (5E1)
C control
D control + anti-Shh mAb (5E1)

Figure S7. N-Shh interaction with Cdh7 is not prevented by anti-Shh mAb (clone 5E1) (related to Figure 4).

(A-D) AP staining of Chd7-expressing **(A, B)** or control **(C, D)** L cells that were incubated with conditioned medium from 293 cells expressing N-Shh-AP in the absence (A, C) or in the presence (B, D) of anti-Shh mAb (clone 5E1). N-Shh-AP signal is detectable in Cdh7-expressing cells regardless of anti-Shh mAb (clone 5E1) treatment, but not in control cells. Scale bar, 100 µm.

Figure S8

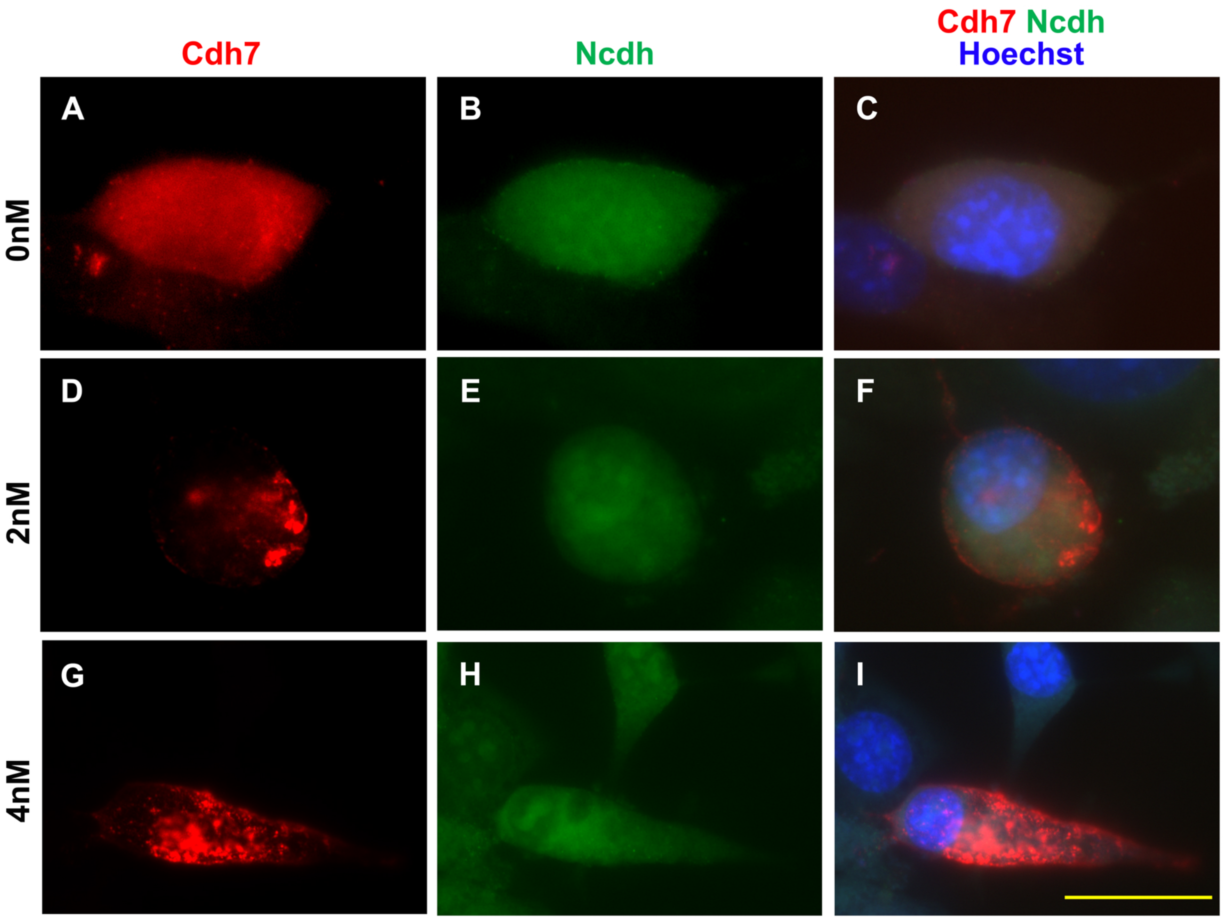

Figure S8. Shh does not promote Ncdh aggregation in NIH3T3 cells (related to Figure 5).

(A-I) Immunofluorescence analysis performed with anti-Cdh7 mAb (red signal) and anti-Ncdh pAb (green signal) using NIH3T3 cells transiently expressing Cdh7 that were cultured for 24 hours in the indicated concentrations of rN-Shh. rN-Shh treatments promote Cdh7 aggregation without altering the distribution of endogenously expressed Ncdh at the cell membrane. Hoechst 33342 staining is shown in blue. Scale bar, 20 µm in **(I)**.

Figure S9

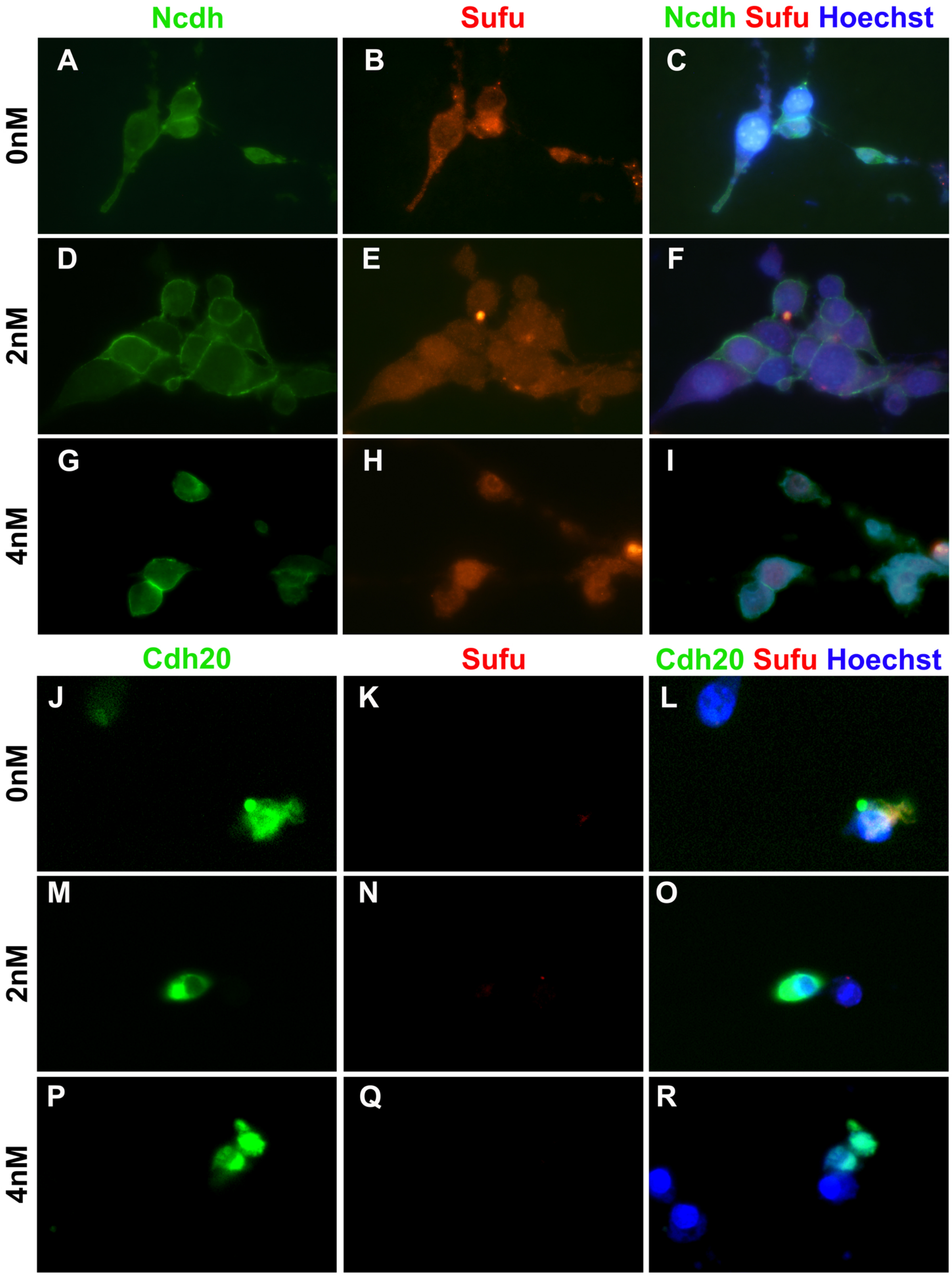

Ncdh
Sufu
Ncdh Sufu Hoechst
0nM
2nM
4nM
A
B
C
D
E
F
G
H
I
Cdh20
Sufu
Cdh20 Sufu Hoechst
0nM
2nM
4nM
J
K
L
M
N
O
P
Q
R

Figure S9. Neither Ncdh nor Cdh20 associate with Sufu in NIH3T3 cells (related to Figure 6).

(A-I) Immunofluorescence analysis performed with anti-Ncdh mAb (green signal) or anti-Sufu pAb (red signal) using NIH3T3 cells transiently expressing Ncdh that were cultured for 24 hours in the indicated concentrations of rN-Shh. No association of Ncdh with Sufu is detectable in the absence or in the presence of rN-Shh. **(C, F, I)** show merging of images in **(A, B)**, **(D, E)**, **(G, H)**, respectively, along with Hoechst 33342 staining (blue signal). Scale bar, 50 µm.

(J-R) Immunofluorescence analysis performed with anti-Myc mAb (green signal) or anti-Sufu pAb (red signal) using NIH3T3 cells transiently expressing Myc-tagged Cdh20 protein that were cultured for 24 hours in the indicated concentrations of rN-Shh. No association of Cdh20 with Sufu is detectable in the presence or in the absence of rN-Shh. **(L, O, R)** show merging of images in **(J, K)**, **(M, N)**, **(P, Q)**, respectively, along with Hoechst 33342 staining (blue signal). Scale bar, 50 µm.

Figure S10

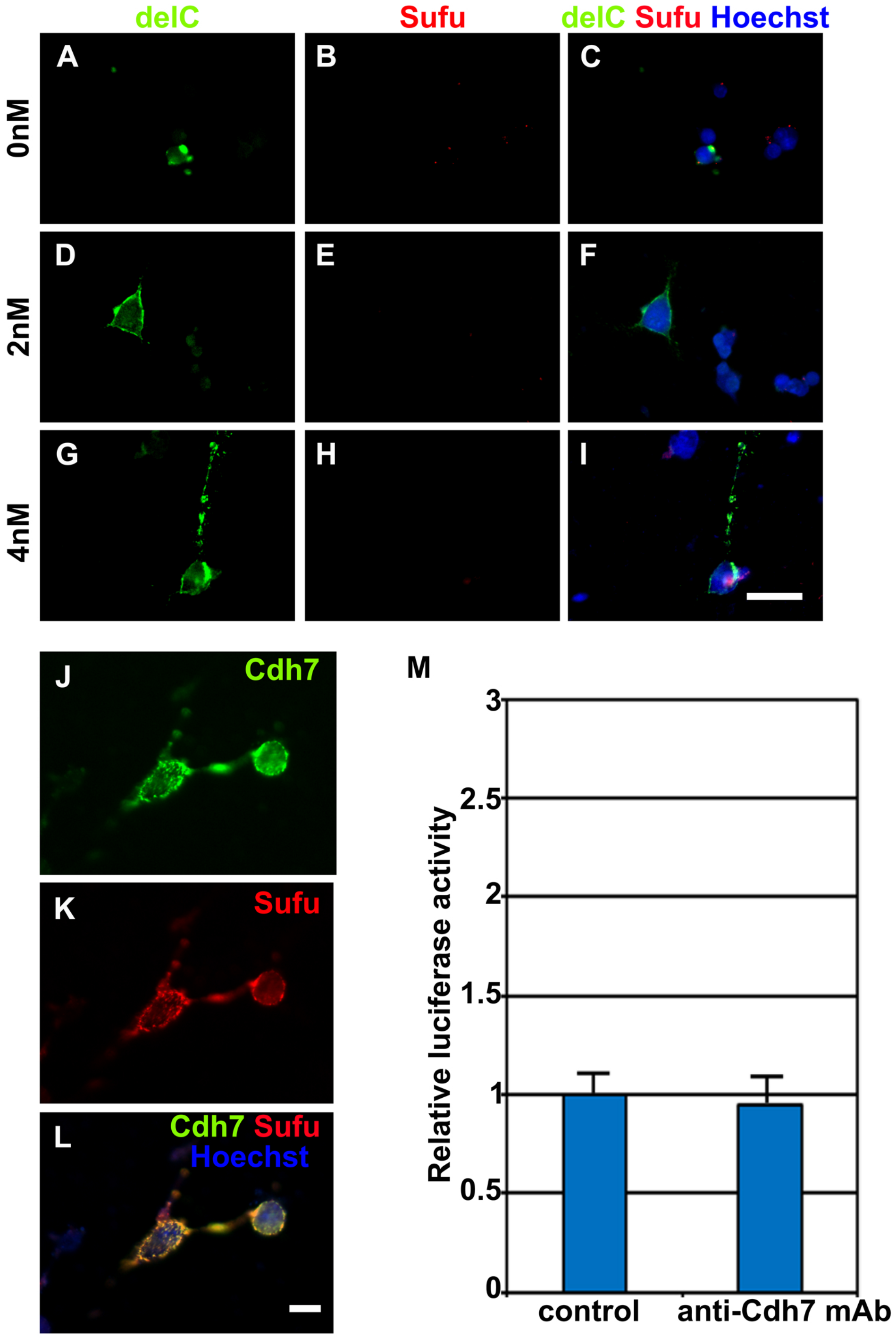
delC
Sufu
delC Sufu Hoechst
A
B
C
0nM
D
E
F
2nM
G
H
I
4nM
J
Cdh7
K
Sufu
L
Cdh7 Sufu
Hoechst
M
Relative luciferase activity
3
2.5
2
1.5
1
0.5
0
control
anti-Cdh7 mAb

Figure S10. Association of Chd7 with Sufu requires Cdh7 cytoplasmic domain (related to Figure 6).

(A-I) Immunofluorescence analysis performed with anti-Cdh7 mAb (green signal) or anti-Sufu pAb (red signal) using NIH3T3 cells transiently expressing delC deletion construct of Cdh7, which were cultured for 24 hours in the indicated concentrations of rN-Shh. No association of delC protein with Sufu is detectable in the absence or in the presence of rN-Shh. **(C, F, I)** show merging of images in **(A, B)**, **(D, E)**, **(G, H)**, respectively, along with Hoechst 33342 staining (blue signal). Scale bar, 50 µm.

(J-L) Immunofluorescence analysis performed with anti-Cdh7 mAb (green signal) or anti-Sufu pAb (red signal) using NIH3T3 cells transiently expressing Cdh7 that were cultured for 4 hours at room temperature in medium containing anti-Cdh7 mAb to cause artificial clustering of Cdh7 molecules. Cdh7 clustering promotes its association with Sufu even in the absence of Shh **(J, K)**. **(L)** show merging of images in **(J)** and **(K)** along with Hoechst 33342 staining (blue signal). Scale bar, 50 µm.

(M) RLA quantification in NIH3T3 cells that were transfected with Cdh7 expression plasmids along with GBS-luc reporter and internal control plasmids, and treated for 4 hours at room temperature with anti-Cdh7 mAb to cause Cdh7 clustering. No RLA

increase is detectable in cells treated with anti-Cdh7 mAb in comparison to untreated cells (control). Results are shown as the mean ± SEM of at least 6 independent experiments.

Figure S11

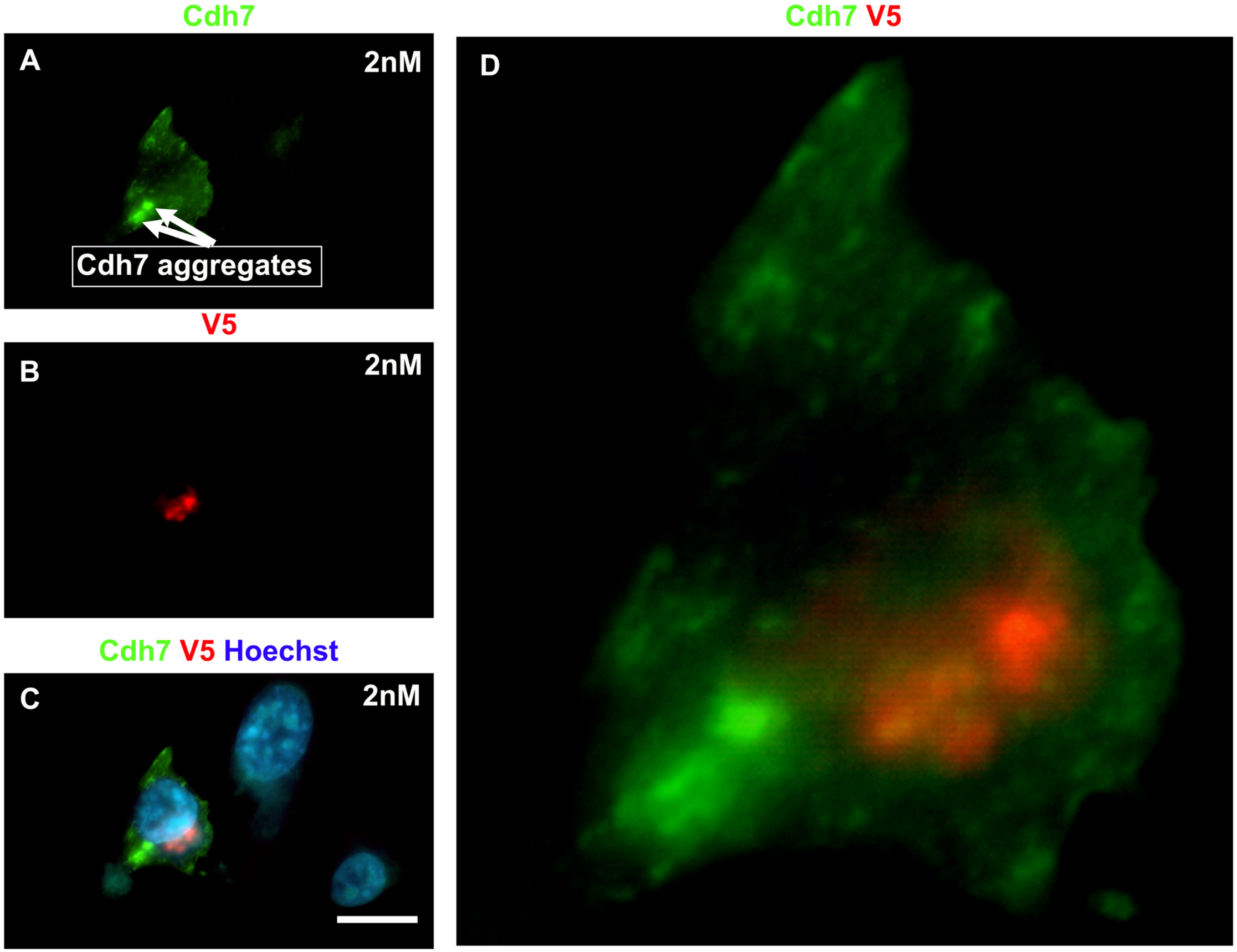
Cdh7
A
2nM
Cdh7 aggregates
V5
B
2nM
Cdh7 V5 Hoechst
C
2nM
Cdh7 V5
D

Figure S11. C-terminal fragments resulting from Gli3FL degradation do not associate with Cdh7 aggregates (related to Figure 6).

(A-D) Immunofluorescence analysis with anti-Cdh7 mAb (green signal) or anti-V5 mAb (red signal) using NIH3T3 cells transiently expressing Cdh7 and a Gli3 chimaeric construct tagged with V5 at the C-terminus, which were treated for 24 hours with 2 nM rN-Shh. rN-Shh causes formation of Cdh7 aggregates at the cell surface (arrow in **(A)**), which do not co-localize with V5 staining. **(C)** show merging of images in **(A)** and **(B)** along with Hoechst 33342 staining (blue signal). **(D)** shows a high magnification image of the $Cdh7^{+}/V5^{+}$ cell in **(A-C)**. Scale bar, 50 µm.

Figure S12

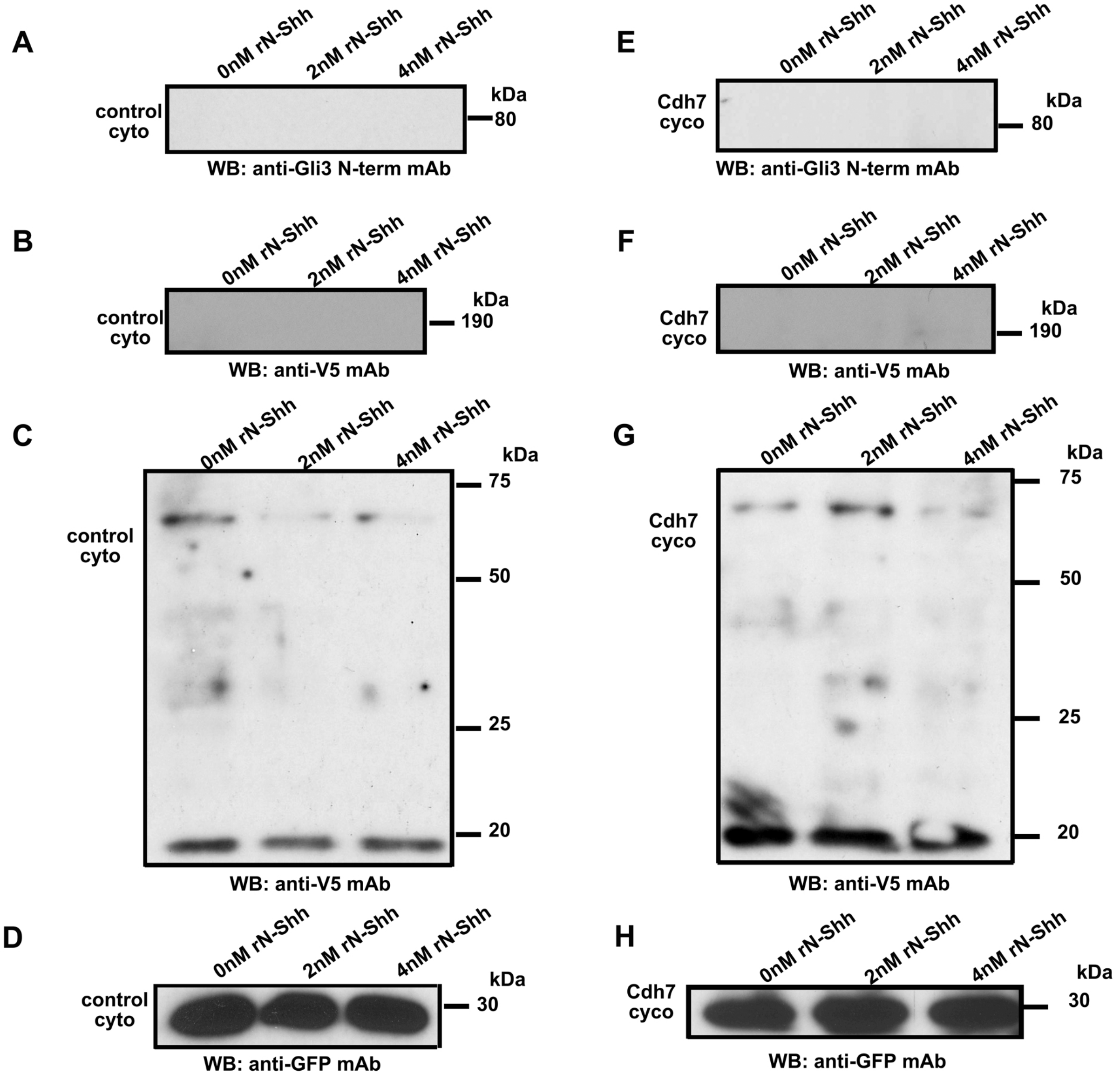
A
0nM rN-Shh
2nM rN-Shh
4nM rN-Shh
control cyto
kDa
80
WB: anti-Gli3 N-term mAb
B
control cyto
kDa
190
WB: anti-V5 mAb
C
control cyto
kDa
75
50
25
20
WB: anti-V5 mAb
D
control cyto
kDa
30
WB: anti-GFP mAb
E
Cdh7 cyco
WB: anti-Gli3 N-term mAb
F
Cdh7 cyco
WB: anti-V5 mAb
G
Cdh7 cyco
WB: anti-V5 mAb
H
Cdh7 cyco
WB: anti-GFP mAb

Figure S12. Cytoplasmic Gli3FL undergoes partial degradation generating C-terminal fragments (related to Figure 6).

(A-H) Immunoblotting analysis using monoclonal antibodies against Gli3 N-terminal region **(A, E)** or V5 **(B, C, F, G)** performed with cytoplasmic extracts of NIH3T3 cells transiently expressing a Gli3 construct tagged with V5 at the C-terminus. Blots in **(A, E)** show 80 kDa Gli3R band, blots in **(B, F)** show 190 kDa Gli3FL band while Gli3 C-terminal fragments between 20 and 75 kDa are shown in **(C, G)**. Control **(A-D)** or Cdh7-expressing cells **(E-H)** were treated with the indicated concentrations of rN-Shh. In the absence or in the presence of rN-Shh, cytoplasmic extracts from both control and Cdh7-expressing cells show the presence of V5-tagged 20-70 kDa fragments, but not 80 kDa Gli3 N-terminus-positive band or 190 kDa V5-positive band, suggesting that Gli3FL undergoes partial proteolysis to C-terminal fragments in these conditions. Transfection efficiency in each condition is shown by immunoblotting using anti-GFP mAb **(D, H)**, following co-transfection of a GFP control plasmid.

Figure S13

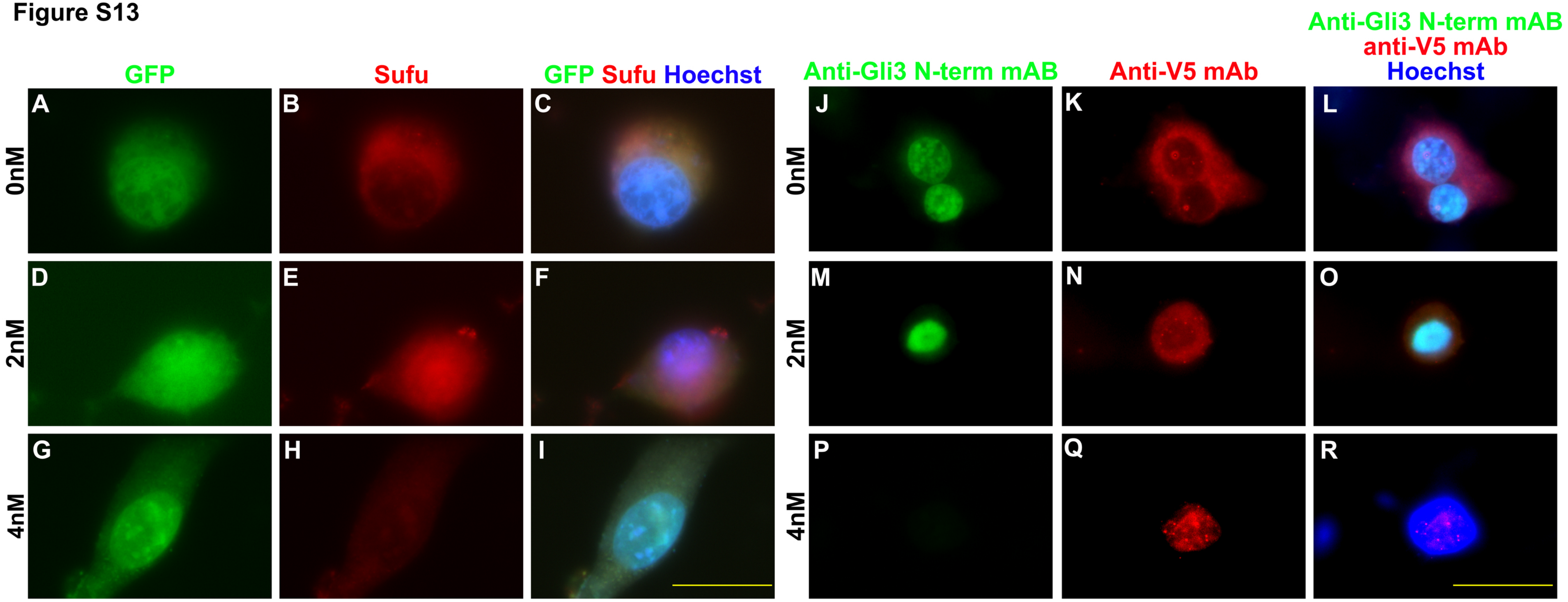

GFP
Sufu
GFP Sufu Hoechst
Anti-Gli3 N-term mAB
Anti-V5 mAb
Anti-Gli3 N-term mAB anti-V5 mAb Hoechst
0nM
2nM
4nM
A
B
C
D
E
F
G
H
I
J
K
L
M
N
O
P
Q
R

Figure S13. Cdh7-targeting siRNA inhibits Cdh7-dependent changes in Sufu localization and Gli3R formation in NIH3T3 cells (related to Figure 6).

(A-I) Immunostaining with anti-Cdh7 (green signal) or anti-Sufu (red signal) Abs using NIH3T3 cells transiently expressing Cdh7 and siRNA-*Cdh7* that were cultured for 24 hours in the indicated concentrations of rN-Shh. When siRNA-*Cdh7* is co-expressed with Cdh7, no major changes in Sufu localization are detectable upon rN-Shh treatments. Scale bar, 40 µm in **(I)**.

(J-R) Immunostaining with anti-GFP (green signal) or anti-V5 (red signal) Abs using NIH3T3 cells transiently expressing a Gli3 chimeric construct tagged with GFP at the N-terminus and V5 at the C-terminus, along with Cdh7 and siRNA-*Cdh7*, and thatwere treated for 18 hours with the indicated concentrations of rN-Shh. When siRNA-*Cdh7* is co-expressed with Cdh7, similar GFP nuclear staining is detectable in untreated cells **(J)** and in cells treated with 2 nM rN-Shh **(M)**, indicating that Cdh7 expression is necessary to prevent Gli3R formation in the presence of low/moderate doses of rN-Shh. No nuclear GFP staining is detectable when cells co-expressing Cdh7 and siRNA-*Cdh7* are treated with 4 nM rN-Shh (4 nM), suggesting that higher doses of rN-Shh

can prevent Gli3R formation independently of Cdh7Scale bar, 40 µm.

Supplementary tables

Table 1. The results of student t-test between each bars in Figure 3. The data of [control, 0nM Shh] and [control, 2nM Shh] (green) are common to each list.

Figure 3A

	control 0nM Shh	control 2nM Shh	control 4nM Shh	Cdh7 0nM Shh	Cdh7 2nM Shh	Cdh7 4nM Shh
control 0nM Shh		0.053099	0.00000477	0.969678	4.89E-09	0.0000299
control 2nM Shh			0.003899	0.0398007	2.23E-06	0.003595
control 4nM Shh				1.15E-06	0.000232	0.48411
Cdh7 0nM Shh					5.24E-09	0.0000252
Cdh7 2nM Shh						0.006843
Cdh7 4nM Shh						

Figure 3B

	control 0nM Shh siRNA-*Cdh7*	control 2nM Shh siRNA-*Cdh7*	control 4nM Shh siRNA-*Cdh7*	Cdh7 0nM Shh siRNA-*Cdh7*	Cdh7 2nM Shh siRNA-*Cdh7*	Cdh7 4nM Shh siRNA-*Cdh7*
control 0nM Shh siRNA-*Cdh7*		0.724991	0.001036	0.353794	0.544584	0.00039
control 2nM Shh siRNA-*Cdh7*			3.23E-05	0.053404	0.242467	5.05E-05
control 4nM Shh siRNA-*Cdh7*				0.000505	0.000957	0.164832
Cdh7 0nM Shh siRNA-*Cdh7*					0.714634	0.001098
Cdh7 2nM Shh siRNA-*Cdh7*						0.002713
Cdh7 4nM Shh siRNA-*Cdh7*						

Figure 3D

	control 0nM Shh	control 2nM Shh	delC 0nM Shh	delC 2nM Shh	delN 0nM Shh	delN 2nM Shh
control 0nM Shh		0.54234	0.56849	0.666941	0.214771	0.358233
control 2nM Shh			0.777164	0.220687	0.025793	0.107702
delC 0nM Shh				0.929821	0.505107	0.649692
delC 2nM Shh					0.489086	0.617065
delN 0nM Shh						0.928524
delN 2nM Shh						

Figure 3E

	control 0nM Shh	control 2nM Shh	Ncdh 0nM Shh	Ncdh 2nM Shh	Cdh20 0nM Shh	Cdh20 2nM Shh
control 0nM Shh		0.882612	0.507591	0.487445	0.756731	0.409545
control 2nM Shh			0.311425	0.179652	0.325489	0.090232
Ncdh 0nM Shh				0.515982	0.288479	0.112104
Ncdh 2nM Shh					0.595912	0.836091
Cdh20 0nM Shh						0.527445
Cdh20 2nM Shh						

Figure 3F

	control DMSO 0nM Shh	control DMSO 2nM Shh	Cdh7 DMSO 0nM Shh	Cdh7 DMSO 2nM Shh	control cyclopamine 0nM Shh	control cyclopamine 2nM Shh	Cdh7 cyclopamine 0nM Shh	Cdh7 cyclopamine 2nM Shh
control DMSO 0nM Shh		0.096185	0.805507	3.1632E-07	1	0.1621	0.677532	0.591931
control DMSO 2nM Shh			0.767403	0.000000573	0.630943	0.136998	0.065641	0.483632
Cdh7 DMSO 0nM Shh				2.8224E-07	0.837352	0.192471	0.570411	0.822494
Cdh7 DMSO 2nM Shh					2.48E-07	0.000000194	2.08E-07	3.66E-07
control cyclopamine 0nM Shh						0.310581	0.737163	0.664215
control cyclopamine 2nM Shh							0.450904	0.093897
Cdh7 cyclopamine 0nM Shh								0.565262
Cdh7 cyclopamine 2nM Shh								

Figure 3G

	control<br>0nM Shh	control<br>2nM Shh	SmoM2<br>0nM Shh	SmoM2<br>2nM Shh	SmoM2<br>Cdh7<br>0nM Shh	SmoM2<br>Cdh7<br>2nM Shh
control 0nM Shh		0.508434	0.0000236	0.0000117	1.15E-07	9.26E-07
control 2nM Shh			1.93E-05	0.000102	1.1E-07	9.11E-07
SmoM2 0nM Shh				0.477989	0.015308	3.61E-06
SmoM2 2nM Shh					0.129939	7.79E-05
SmoM2 Cdh7 0nM Shh						5.27E-05
SmoM2 Cdh7 2nM Shh						

Figure 3H

	control<br>0nM Shh	control<br>2nM Shh	Gli3FL<br>0nM Shh	Gli3FL<br>2nM Shh	Gli3FL<br>Cdh7<br>0nM Shh	Gli3FL<br>Cdh7<br>2nM Shh
control 0nM Shh		0.508434	0.0000526	8.46E-09	0.00072	9.18E-09
control 2nM Shh			4.43E-05	8E-09	0.0006	8.6E-09
Gli3FL 0nM Shh				0.577751	0.248668	0.000152
Gli3FL 2nM Shh					0.367691	0.00000942
Gli3FL Cdh7 0nM Shh						0.0000162
Gli3FL Cdh7 2nM Shh						

Table 2. The results of student t-test between each bars in Figure S3. The data of [control, 0nM Shh] and [control, 2nM Shh] (green) are common to each list.

Figure S3A

	control 0nM Shh	control 2nM Shh	Gli1 0nM Shh	Gli1 2nM Shh	Gli1 Cdh7 0nM Shh	Gli1 Cdh7 2nM Shh
control 0nM Shh		0.508434	4.47E-07	0.0000674	9.32E-08	0.0000128
control 2nM Shh			4.46E-07	6.73E-05	9.31E-08	1.28E-05
Gli1 0nM Shh				0.897642	0.113317	0.028525
Gli1 2nM Shh					0.24115	0.065314
Gli1 Cdh7 0nM Shh						0.345458
Gli1 Cdh7 2nM Shh						

Figure S3B

	control 0nM Shh	control 2nM Shh	Gli2 0nM Shh	Gli2 2nM Shh	Gli2 Cdh7 0nM Shh	Gli2 Cdh7 2nM Shh
control 0nM Shh		0.508434	0.0000606	4.53E-08	0.00000465	0.001483
control 2nM Shh			6.1416E-05	4.62E-08	0.0000047	0.001496
Gli2 0nM Shh				0.905524	0.140262	0.77004
Gli2 2nM Shh					0.086574	0.811935
Gli2 Cdh7 0nM Shh						0.367064
Gli2 Cdh7 2nM Shh						

Figure S3C

	control 0nM Shh	control 2nM Shh	Gli3R 0nM Shh	Gli3R 2nM Shh	Gli3R Cdh7 0nM Shh	Gli3R Cdh7 2nM Shh
control 0nM Shh		0.508434	0.755012	0.890642	0.058548	0.034407
control 2nM Shh			0.418357	0.595106	0.014188	0.007385
Gli3R 0nM Shh				0.673968	0.199253	0.135654
Gli3R 2nM Shh					0.043456	0.024979
Gli3R Cdh7 0nM Shh						0.776821
Gli3R Cdh7 2nM Shh						

Table 3. The results of student t-test between each bars in Figure S4.

Figure S4A

	control 0nM Shh	control 2nM Shh	control 4nM Shh	Cdh7 0nM Shh	Cdh7 2nM Shh	Cdh7 4nM Shh
control 0nM Shh		0.396026	0.000558	0.781537	0.002686	0.000453
control 2nM Shh			0.003348	0.509217	0.004729	0.00116
control 4nM Shh				0.000671	0.054137	0.059667
Cdh7 0nM Shh					0.003315	0.000595
Cdh7 2nM Shh						0.6285
Cdh7 4nM Shh						

Figure S4B

	control 0nM Shh siRNA-*Cdh7*	control 2nM Shh siRNA-*Cdh7*	Cdh7 0nM Shh siRNA-*Cdh7*	Cdh7 2nM Shh siRNA-*Cdh7*
control 0nM Shh siRNA-*Cdh7*		0.28120481	0.495702	0.230151
control 2nM Shh siRNA-*Cdh7*			0.746834	0.630528
Cdh7 0nM Shh siRNA-*Cdh7*				0.544481
Cdh7 2nM Shh siRNA-*Cdh7*				

Figure S4C

	control 0nM Shh	control 2nM Shh	Cdh7 0nM Shh	Cdh7 2nM Shh	delC 0nM Shh	delC 2nM Shh	delN 0nM Shh	delN 2nM Shh
control 0nM Shh		0.329499	0.63988	0.00016	0.361135	0.035106	0.745933	0.984202
control 2nM Shh			0.21414	0.000131	0.924957	0.515803	0.986392	0.339821
Cdh7 0nM Shh				0.000158	0.230307	0.014912	0.986392	0.690562
Cdh7 2nM Shh					0.000136	0.000192	0.000118	0.000151
delC 0nM Shh						0.430819	0.311766	0.372886
delC 2nM Shh							0.063124	0.046032
delN 0nM Shh								0.768232
delN 2nM Shh								

Figure S4D

	control 0nM Shh	control 2nM Shh	Cdh7 0nM Shh	Cdh7 2nM Shh	Cdh20 0nM Shh	Cdh20 2nM Shh
control 0nM Shh		0.924172	0.596179	0.000915	0.33449	0.87309
control 2nM Shh			0.676143	0.000876	0.500508	0.816632
Cdh7 0nM Shh				0.000867	0.957899	0.513711
Cdh7 2nM Shh					0.001049	0.000927
Cdh20 0nM Shh						0.191227
Cdh20 2nM Shh						

Figure S4E

	control DMSO 0nM Shh	control DMSO 2nM Shh	Cdh7 DMSO 0nM Shh	Cdh7 DMSO 2nM Shh	control cyclopamine 0nM Shh	control cyclopamine 2nM Shh	Cdh7 cyclopamine 0nM Shh	Cdh7 cyclopamine 2nM Shh
control DMSO 0nM Shh		0.954642	0.065321	3.5357E-05	0.672950347	0.73564	0.648388	0.409602985
control DMSO 2nM Shh			0.348637	7.1347E-06	0.692822609	0.805305	0.651627	0.418942559
Cdh7 DMSO 0nM Shh				3.2148E-05	0.832899437	0.318016	0.946164	0.63514386
Cdh7 DMSO 2nM Shh					1.00514E-06	1.36E-06	8.14E-07	4.48185E-06
control cyclopamine 0nM Shh						0.581694	0.862429	0.595575758
control cyclopamine 2nM Shh							0.568939	0.366394177
Cdh7 cyclopamine 0nM Shh								0.75589215
Cdh7 cyclopamine 2nM Shh								

Figure S4F

		SmoM2 Cdh7 2nM Shh	Gli1 Cdh7 2nM Shh	Gli2 Cdh7 2nM Shh	Gli3FL Cdh7 2nM Shh	Gli3R Cdh7 2nM Shh
SmoM2	2nM Shh	3.597E-05				
Gli1	2nM Shh		0.20591			
Gli2	2nM Shh			0.52891		
Gli3EL	2nM Shh				1.25117E-06	
Gl3R	2nM Shh					0.52648

Table 4. The results of student t-test between each bars in Figure S5.

Figure S5A

without siRNA-*Cdh7*	control	control + cShh	Cdh7	cdh7 + cShh
control		1.10403E-05	0.00528	0.000221
control + cShh			0.00428	0.000795
cdh7				0.000489

With siRNA-*Cdh7*	control	control + cShh	Cdh7	Cdh7 + cShh
control + siRNA -*Cdh7*	0.291167			
control + cShh + siRNA-*Cdh7*		0.337177106		
Cdh7 + siRNA -*Cdh7*			0.49689	
Cdh7 + cShh + siRNA-*Cdh7*				0.000726

Figure S5C

- cShh	Cdh7	delC	delN
control	0.63988	0.00736	0.00580

+ cShh	Cdh7 + cShh	delC + cShh	delN + cShh
control + cShh	0.0019644	0.181868425	0.068353

Figure S5D

- cShh	Cdh7	Cdh20
control	0.53467	0.013

+ cShh	Cdh7 + cShh	Cdh20 + cShh
control + cShh	0.000474	0.079081

Figure S5E

DMSO	control + cShh	Cdh7	Cdh7 + cShh
control	0.001188	0.007949836	0.0000354
control + cShh		0.557776	0.0000839
Cdh7			4.34E-05

Cyclopamine-KADD	control	control + cShh	Cdh7	Cdh7 + cShh
control		0.946416936	0.395719	0.255835
control + cShh			0.599063	0.478228
Cdh7				0.686187

Figure S5F

	SmoM2 + cShh + Cdh7
SmoM2 + cShh	0.00000843

Figure S5G

	Gli1 + cShh + Cdh7
Gli1 + cShh	0.611268

Figure S5H

	Gli2 + cShh + Cdh7
Gli2 + cShh	0.824024

Figure S5I

	Gli3FL + cShh + Cdh7
Gli3FL + cShh	0.000000126

Figure S5J

	Gli3R + cShh + Cdh7
Gli3R + cShh	0.381108

Table 5. The ratio of density of pull downed band and input band (related to Figure 4). The data were standardized with a value of 0nM rN-Shh.

Figure 4I

WB: anti-Cdh7 mAb	0nM rN-Shh	2nM rN-Shh	4nM rN-Shh
Pull down / Input	1	0.902479	1.111111

Figure 4J

WB: anti-Gli3FL pAb	0nM rN-Shh	2nM rN-Shh	4nM rN-Shh
Pull down / Input	1	2.007871	1.670826

Figure 4K

WB: anti-Sufu pAb	0nM rN-Shh	2nM rN-Shh	4nM rN-Shh
Pull down / Input	1	2.126843	2.003795

Figure 4L

WB: anti-Gli3R pAb	0nM rN-Shh	2nM rN-Shh	4nM rN-Shh
Pull down / Input	1	0.872593	0.926489

Figure 4M

WB: anti-GSK3β pAb	0nM rN-Shh	2nM rN-Shh	4nM rN-Shh
Pull down / Input	1	0.935949	0.979804

Table 6. The ratio of density of pull downed band and input band (related to Figure 6). The data were standardized with a value of 0nM rN-Shh.

Figure 6AT/AV (control, nuc)

WB: anti-Gli3R mAb	0nM rN-Shh	2nM rN-Shh	4nM rN-Shh
Pull down / Input	1	1.675239	0.556971

Figure 6AU/AV (control, nuc)

WB: anti-Gli3FL pAb	0nM rN-Shh	2nM rN-Shh	4nM rN-Shh
Pull down / Input	1	1.917001	2.128062

Figure 6AW/AY (Cdh7, nuc)

WB: anti-Gli3R mAb	0nM rN-Shh	2nM rN-Shh	4nM rN-Shh
Pull down / Input	1	0.554552	0.527586

Figure 6AX/AY (Cdh7, nuc)

WB: anti-Gli3FL pAb	0nM rN-Shh	2nM rN-Shh	4nM rN-Shh
Pull down / Input	1	0.935	0.977778

Supplementary Materials and Methods

Animals

Fertilized eggs of White Leghorn chickens were purchased from a commercial source (Yamagishi Farm, Nagoya, Japan) and incubated in a humidified chamber at 38°C. Embryo staging was performed as previously described (Hamburger and Hamilton, 1992).

Generation of DNA constructs

Chick *Cdh7* from pCAGGS-*Cdh7* plasmid (Kawano et al., 2002) was subcloned into pCAGGS vector in frame with a carboxyl terminal 6xmyc epitope-tagged fragment (pCAGGS-*Cdh7*-6x-myc), which was generated by restriction digestion of pCS2+MT**.** For electroporation into chick embryos, *Cdh7*-6x-myc insert from pCAGGS-*Cdh7*-6x-myc plasmid was subcloned into pCIG vector (Megason and McMahorn, 2002) (pCIG-*Cdh7*-6x-myc).

Cdh7 N-terminal deletion constructs CR3, CR4, or preTM were generated by fusing Cdh7 signal peptide to amino acid residues 291, 405, or 510 of Cdh7-6x-myc. The del-preTM-6x-myc construct was generated by deleting the preTM domain of Cdh7-6x-myc. The del-CR4-preTM-6x-myc construct was generated by deleting the CR4 and preTM domains of Cdh7-6x-myc. All constructs were generated by conventional PCR using pCAGGS-*Cdh7*-6x-myc or pCIG-*Cdh7*-6x-myc as templates with the following forward (F) and reverse (R) primers:

Cdh7-CR3-F (5’-GTGGGACCCAATGCTGAAATGG-3’),

Cdh7-CR4-F (5’-GCCTCAAATAGTCCAGTCAGGTAC-3’),

Cdh7-preTM-F (5’-GACCCACCAAATGGTCATCAGTTCTAC-3’),

Cdh7-TM-F (5’-GGAGCCCTGATAGCAATATTGGCTTG-3’),

Cdh7-n-signal-R (5’-GCGTTTGGTTCTCGTCCTCCCAGACTG-3’),

Cdh7-del CR4-R (5’-ATCTGGATCATGGGCAGCAACAGTTCC-3’),

Cdh7-del-preTM-R (5’-ATCTTTATCAATTGCACTGATTTTCTGGATGATC-3’).

Cdh7 delC construct includes the extracellular, transmembrane and the first 15 amino acids of the cytoplasmic domains of Cdh7-6x-myc, excluding the β-catenin binding site. This construct was produced by ligating a DraI 6x-myc epitope-tagged fragment into the EcoRV site of pCAGGS-*Cdh7*-6x-myc. Cdh7 delN construct was fused to the signal peptide of chick Cdh7 at amino acid residue 608 of Cdh7-6x-myc. This construct was generated by PCR with the above-described primers Cdh7-TM-F and Cdh7-n-signal-R using pCAGGS-*Cdh7*-6x-myc as a template.

pMES-*Cdh20*-6x-myc was generated by fusing chick *Cdh20* cDNA (Shirabe et al., 2005) to a carboxyl terminal 6x-myc epitope-tagged fragment, followed by subcloning into pMES (Chen et al., 2004).

For the generation of *Gli3* constructs, total RNA was purified from P0 mouse brain (Isogen, Nippongene) and converted to cDNA by reverse-transcription (SuperScript III, Invitrogen). pcDNA3.1/*mGli3*-V5 was generated by PCR using this cDNA as a template with the following primers:

mGli3-V5-F (5’-CACCATGGAGGCCCAGGCCCACAGCTC-3’) and

mGli3-R (5’-CTGCATAACTGCAAGGAACTTGCTTTCTTC-3’).

The PCR product amplified with these primers was then subcloned using pcDNA3.1 Directional TOPO TA kit (Invitrogen). pcDNA3.1-GFP-*mGli3*-V5 was made by fusing a GFP-encoding fragment from pEGFP-N1 plasmid (Clontech) to the 5'-end of *mGli3*-V5 sequence, followed by subcloning into pcDNA3.1.

Cdh7-targeting siRNA was generated using pSilencer 1.0-U6 System (Ambion), with the target sequence 5’-GATAACACTGCAACTGTAT-3’. The human U6 promoter together with the sequence encoding for *Cdh7*-targeting siRNA were ligated into the pCAGGS2-EGFP vector to generate pCAGGS-siRNA-*Cdh7*-GFP plasmid (siRNA-*Cdh7*) (a kind gift from Dr. Okafuji). U6 promoter together with the sequence encoding for a control siRNA with limited homology to any known sequences in the human, mouse or rat genomes (Ambion) were cloned in the same vector to generate the control pCAGGS-siRNA-*control*-GFP plasmid (siRNA-*control*). Each construct was validated by DNA sequencing using an ABI Prism 310 (Applied Biosystems).

pCAGGS-chick *Shh* was a gift from Dr. C. Tabin, and pAP-tag-5 –N-Shh AP was generated by subcloning the sequence encoding for chick Shh N-terminal region (Williams et al., 1999)into pAP-tag-5 (GenHunter).

pCAGGS-*cNcdh* and pCAGGS-*mNcdh*, encoding for chick and mouse Ncdh, respectively, were gifts from Dr. M. Takeichi. pGE-mSmoM2-EA was purchased from ATCC. GBS-Luc reporter plasmid, pcDNA3.1/HisB-*mGli1*, pcDNA3.1/HisB-*mGli2*, and pcDNA3.1/HisB-*hGli3⊿C* were kind gifts from Dr. H. Sasaki.

Antibodies

Primary antibodies used for this study were mouse IgG_1 monoclonal antibody against chick Cdh7 (clone CCD7-1, a generous gift from Dr. M. Takeichi) (Supernatant from hybridoma cells was used undiluted for immunofluorescence (IF), with a 1/2 dilution for immunoprecipitation (IP), with a 1/10 dilution for Western blot (WB)), mouse IgG_1 monoclonal antibody against chick Pax7 (DSHB, Developmental Studies Hybridoma Bank) (Supernatant used undiluted for IF, 1/2 dilution for IP, 1/10 dilution for WB), mouse IgG monoclonal antibody against chick Isl1/2 (DSHB) (Supernatant used undiluted for IF, 1/2 dilution for IP, 1/10 dilution for WB), mouse IgG monoclonal antibody against chick Lim1/2 (DSHB) (Supernatant used undiluted for IF, 1/2 dilution for IP, 1/10 dilution for WB) mouse IgG_1 monoclonal antibody against chick Shh (clone 5E1, DSHB)

(Supernatant used with a 1/2 dilution for IF, 1/4 dilution for IP, 1/100 dilution for WB), mouse IgG1 monoclonal antibody against mouse Ncdh (32/N-Cadherin, BD Bioscience) (used 1/300 for IF, 1/10 for IP, 1/2000 for WB), rabbit polyclonal antibody against human Ncdh (H-63, Santa Cruz) (used 1/300 for IF, 1/10 for IP, 1/2000 for WB), rabbit polyclonal antibody against chick Shh (Santa Cruz) (used 1/300 for IF, 1/10 for IP, 1/2000 for WB), goat polyclonal antibody against mouse Gli3 (N19, Santa Cruz) (used 1/300 for IF, 1/10 for IP, 1/2000 for WB), rabbit polyclonal antibody against mouse Gli3 (H280, Santa Cruz), (used 1/300 for IF, 1/10 for IP, 1/2000 for WB) rabbit polyclonal antibody against human GLI-Kruppel family member Gli3 (Lifespan Biosciences) (used 1/300 for IF, 1/10 for IP, 1/2000 for WB), rabbit polyclonal antibody against mouse Sufu (Santa Cruz) (used 1/250 for IF, 1/10 for IP, 1/2000 for WB), rabbit polyclonal antibody against human GSK-3β (Abnova) (used 1/300 for IF, 1/10 for IP, 1/2000 for WB), goat polyclonal antibody against human Dbx1 (T14, Santa Cruz) (used 1/50 for IF), mouse IgG_1 monoclonal antibody against Myc (clone 9E10, DSHB**)** (Supernatant used 1/2 for IF, 1/4 for IP, 1/100 WB)**,** rabbit polyclonal antibody against myc (Upstate) (used 1/300 for IF, 1/10 for IP, 1/2000 for WB), mouse IgG_{2a} monoclonal antibody against V5 (Invitrogen) (used 1/300 for IF, 1/10 for IP, 1/2000 for WB), mouse IgG_{2b} monoclonal antibody against GFP (clone 1E4, Medical

& Biological Lab) (used 1/300 for IF, 1/2000 for WB), rabbit monoclonal antibody against β-catenin (D10A8, Cell Signaling) (used 1/300 for IF, 1/10 for IP, 1/2000 for WB). Secondary antibodies used were Alexa 488 and 546 goat anti-mouse IgG_1 (anti-mouse IgG_1 Ab-Alexa 488 or 546) (1/500 for IF), Cy3 goat anti-mouse IgG (H+L) (anti-mouse IgG (H+L) Ab-Cy3) (1/500 for IF), Alexa 488 and 546 goat anti-mouse IgG_{2b} (anti-mouse IgG_{2b} Ab-Alexa 488 or 546) (1/500 for IF), Alexa 488 goat anti-rabbit IgG (H+L) (anti-rabbit IgG_1 Ab-Alexa 488; Molecular Probes) (1/500 for IF), goat anti-mouse IgG (H+L)–HRP (Southern Biotech) (1/5000 for WB), goat anti-mouse IgG_1–AP (Southern Biotech) (1/1000 for IF), Alexa 488 and 546 donkey anti-goat IgG (H+L) (Molecular Probes) (1/300 for IF), goat anti-rabbit IgG (H+L)–HRP (Southern Biotech), (1/5000 for WB) and donkey anti-goat IgG (H+L)-HRP (Southern Biotech) (1/5000 for WB). All antibodies were diluted in PBS containing 10% skim milk at appropriate concentrations.

In ovo electroporation

pCIG-Cdh7-6x-myc (3.5 μg/μl), pCAGGS-Shh (3.5 μg/μl), pCAGGS- siRNA-*Cdh7* (3.5 μg/μl), Cdh7-delC (3.5 μg/μl), Cdh7-

delN (3.5 µg/µl), pEF-1-Cdh7(soluble variant)-6x-myc (3.5 µg/µl), pMES-Cdh20-6x-myc (3.5 µg/µl), pCAGGS-cNcdh (3.5 µg/µl), pGE-mSmo-M2-EA (3.5 µg/µl), empty pCIG (3.5 µg/µl) vector, siRNA-*Cdh7* (3.5 µg/µl) or siRNA-*control* (3.5 µg/µl) plasmids were injected into the spinal cord lumen of HH st. 10 chick embryos. Square pulses (50 mm seconds 25 V, three times, 10 mm second intervals) were applied using a CUY21 electroporator (Neppa Gene). Chick embryos were harvested for end-point analyses at HH st. 15, 17 or 23.

***In situ* hybridization**

Antisense probe for chick *Cdh7* was prepared by subcloning a 3,000 bp *Cdh7* cDNA into pGEM-T easy vector (Promega), followed by in vitro transcription of digoxigenin-labelled (Roche) antisense RNA. *Cdh7* probe was hydrolyzed and used for *in situ* hybridization.

Chick embryos at HH st. 17 and 23 were fixed in 4% paraformaldehyde (PFA)/PBS at 4°C overnight and washed in PBS at 4°C for 5 hours. Embryos were cryoprotected overnight in 15% (w/v) sucrose in PBS, embedded in Tissue-Tek OCT compound

(Sakura) and frozen at -80°C for 1 hour. In total, 20 µm of sections were cut on a cryostat and collected on MAS-coated glass slides (Matsunami). Section *In situ* hybridization on chick embryo sections was performed as previously described (Schaeren-Wiemers and Gefin-Moser, 1993). Hybridized samples were mounted with Entellan (Merck) and examined under a Nikon microscope. Images were captured using CCD image sensor (E600 Nikon) and processed with Adobe Photoshop 7.0.

Immunohistochemistry

Chick embryos were fixed in 4% PFA/PBS at 4°C for 1 hour, washed in PBS at 4°C for 5 hours and cryoprotected overnight in 15% (w/v) sucrose in PBS, followed by. embedding in Tissue-Tek OCT compound (Sakura) and freezing at -80°C for 1 hour. In total, 14 µm of sections were cut on a cryostat and collected on MAS-coated glass slides (Matsunami).

Section immunostaining: Embryo sections were incubated in conditioned medium (RPMI 1640 with 10% FCS) with anti-Pax7 mAb, anti-Dbx1 pAb, anti-Cdh7 mAb, anti-Shh mAb, anti-Isl1/2 mAb, , anti-Lim1/2 mAb, anti-Ncdh mAb, anti-myc mAb,, and/or anti-GFP mAb Sections were washed with PBS and incubated in 5% skim milk containing secondary antibodies for

1 hour at room temperature (RT), followed by washing with PBS, incubation with Hoechst 33342 (in PBS, 1/1000) for 5 minutes at RT and mounting in PBS containing 90% glycerol and 1% propyl gallate.

Preparation of conditioned media

For preparation of conditioned media containing Shh or N-Shh-AP, 293 cells were cultured with DMEM and 10% FCS in 10-cm diameter tissue culture dishes (Nunc) and transfected with 24 µg of pCAGGS-*Shh*, pAP-tag-5–N-Shh- or mock vector using Lipofectamine 2000 (Invitrogen). Conditioned media were collected after 5 to 7 days of cell culture.

Immunoprecipitation

For immunoprecipitation assay to confirm binding of Cdh7 to Shh, transient Cdh7-expressing NIH3T3 cells or control NIH3T3 cells were cultured in 10-cm diameter tissue culture dishes (Nunc). Semi-confluent cells were harvested in lysis buffer (50 mM HEPES [pH 7.5], 150 mM NaCl, 5 mM $CaCl_2$, 1% Triton X-100, 1 mM PMSF, 10 µg/ml aprotinin, 10 µg/ml leupeptin), and

incubated with rN-Shh (0.2ng/ml) overnight at 4℃.

For immunoprecipitation assays to verify binding of Cdh20 or Ncdh to Shh, NIH3T3 cells were cultured in 10-cm diameter tissue culture dishes (Nunc) and transfected with 24 µg of pMES-*Cdh20*-6x-myc, or pCAGGS-*cNcdh* using Lipofectamine 2000 (Invitrogen). After 48 hours, cells were harvested using lysis buffer, and incubated with rN-Shh (0.2ng/ml) overnight at 4℃.

For immunoprecipitation assays to verify of Cdh7 to Shh endogenously, HH st.17 embryos were homogenized in lysis buffer and incubated overnight at 4℃.

Input samples were prepared by diluting 100 µl of lysate with an equal volume of 2x Laemmli sample buffer (20 mM Tris-HCl [pH 8.0], 2 mM EDTA, 2% SDS, 20% sucrose, 1.2% mercaptoethanol, and 0.01% BPB), followed by centrifugation at 15,000 rpm for 20min at 4℃. Supernatants were incubated with different antibodies (anti-Cdh7 mAb, anti-Shh mAb, anti-myc mAb, or anti-Ncdh pAb overnight at 4°C on a rotator.

For immunoprecipitation assay investigating binding of Cdh7 to components of Shh signaling pathway, NIH3T3 cells were

cultured in 10-cm diameter tissue culture dishes (Nunc) and transfected with 24 µg of pCAGGS-*Cdh7* using Lipofectamine LTX (Invitrogen). After 24 hours, cells were treated with different concentrations of recombinant mouse Shh amino-terminal peptide (rN-Shh; R&D Systems) in OPTI-MEM (Invitrogen) and harvested with lysis buffer, followed by immmunoprecipitation with anti-Cdh7 mAb as described above.

For immunoprecipitation assays to verify binding of Cdh7, Cdh20 or Ncdh to Shh in the embryonic spinal cord *in vivo*, 10 chick HH st. 17 spinal cords were homogenized using a 22G needle in lysis buffer. After centrifugation at 14,000 rpm for 20 minutes at 4°C, the supernatant was processed for immunopecipitation with Cdh7, Ncdh or Shh antibodies as described above.

To identify Cdh7 binding site for Shh, COS-7 cells were cultured in 10-cm diameter tissue culture dishes (Nunc) and transfected with 24 µg of pCAGGS-*Cdh7* full-length, CR3, CR4, preTM, del-CR4-preTM, del-preTM, delN or delC constructs using Lipofectamine LTX (Invitrogen). After 48 hours, cells were harvested in lysis buffer and processed as described above for immunoprecipitation with anti-Shh mAb.

For all these immunoprecipitation assays, following antibody binding, 100 µl Protein G-Sepharose beads were added to each sample, followed by incubation for 1 hour at 4°C on a rotator. Beads were washed three times with lysis buffer and one time with 20 mM Tris-HCl [pH 7.0], resuspended in 100 µl 2x Laemmli sample buffer, and heated at 95°C for 5 minutes before supernatants were collected. Immunoprecipitated proteins were resolved by SDS-PAGE, transferred to PVDF membranes, followed by immunoblotting with the desired antibodies. Quantification of band intensity was performed using NIH Image and Photoshop CS4 softwares.

AP binding assay

Control COS-7 cells or COS-7 cells transfected with pCAGGS-*Cdh7* or pCAGGS-*cNcdh* were incubated in conditioned medium containing N-Shh-AP. Bound N-Shh-AP protein was visualized with BM purple AP substrate (Roche) for cell surface staining or with AP yellow liquid substrate (Sigma) to quantify N-Shh-AP binding in cell extracts. Kd measurements were determined by nonlinear regression analysis of saturation binding data.

Explant cultures

Explants of presumptive intermediate spinal cord were dissected from HH st. 10 chick embryos and cultured in RPMI 1640 with or without rN-Shh or SAG (3-Chloro-N-[trans-4-(methylamino)cyclohexyl]-N-[3-(4-pyridinyl)benzyl]-1-benzothiophene-2-carboxamide) (Adipogen life science) for 24 hours, then fixed in 4% PFA/PBS for 5 minutes at RT. Immunostaining was performed by incubation of fixed explants in conditioned medium (RPMI 1640 with 10% FCS) from hybridomas producing anti-Islet1/2 mAb, anti-Lim1/2 mAb, anti-Pax7 mAb or anti-Cdh7 mAb for 1 hour at RT, followed by washes with PBS and incubation in 5% skim milk containing anti-mouse IgG (H+L)-Alexa-488 or anti-mouse IgG-Cy3 for 1 hour at RT. Explants were then washed with PBS, stained using Hoechst 33342 for 5 min at RT, mounted in PBS containing 90% glycerol and 1% propyl gallate, and imaged with a Nikon fluorescence microscope and a CCD image sensor (E600 Nikon).

In ovo luciferase assays

In ovo luciferase assays with GBS–Luc reporter plasmid (Sasaki et al., 1997) were performed using the Dual Luciferase Reporter Assay System (Promega). HH st. 10 chick embryos were electroporated with a plasmid mixture including Cdh7 expression plasmid (pCAGGS-*Cdh7*, 3.5 μg/μl), GBS-luc reporter plasmid (3.5 μg/μl) and an internal control plasmid (pRL-tk, Promega, 1.0 μg/μl) *in ovo* (square pulses were 50 mm seconds 25 V, three times, 10 mm second intervals).Electroporated embryos were incubated for 6 hours, followed by dissection of presumptive intermediate spinal cord explants. Explants were cultured in 1 ml RPMI 1640 with or without rN-Shh, DMSO (Wako) or 25nM cyclopamine-KAAD (Toronto Research Chemicals) for 24 hours, then harvested for detection of relative luciferase activity as previously described (Dessaud et al., 2007).

***In vitro* luciferase assays**

NIH3T3 cells were cultured in 6-well tissue culture plates (Nunc) and transiently transfected using FuGENE 6 reagent (Roche). After 24 hours, cells were transfected with 1 μg of the plasmid mixture described above. The following day, cell culture media were changed with 2 ml/well of RPMI 1640 with or without rN-Shh, DMSO or 25nM cyclopamine-KAAD. Luciferase assays

were performed 24 hours later using the Dual Luciferase Reporter Assay System.

The following plasmid weight (µg) ratios were used for co-electroporation or transfection experiments:

Figure 3A, 3F, S4A, S4C, S4D and S4E (control assays): pCAGGS:GBS-luc:pRL-tk (20:10:1).s of F

Figure 3A, 3F, S4A, S4C, S4D and S4E (Cdh7 expression assays): pCAGGS-*Cdh7*:pCAGGS:GBS-luc:pRL-tk (10:10:10:1).

Figure 3B and S4B (siRNA-*Cdh7* expression assays): pCAGGS:pCAGGS- siRNA-*Cdh7*:GBS-luc:pRL-tk (10:10:10:1)

Figure 3B and S4B (Cdh7+ siRNA-*Cdh7* expression assays): pCAGGS-*Cdh7*:pCAGGS- siRNA-*Cdh7*:GBS-luc:pRL-tk (10:10:10:1)

Figure 3D and S4C (Cdh7-delC expression assays): *Cdh7-delC*:pCAGGS:GBS-luc:pRL-tk (10:10:10:1).

Figure 3D and S4C (Cdh7-delN expression assays): *Cdh7-delN*:pCAGGS:GBS-luc:pRL-tk (10:10:10:1).

Figure 3E (Ncdh expression assays): *Ncdh*:pCAGGS:GBS-luc:pRL-tk (10:10:10:1).

Figure 3E and S4D (Cdh20 expression assays): *cCdh20*:pCAGGS:GBS-luc:pRL-tk (10:10:10:1).

Figure 3G (SmoM2 expression assays): *SmoM2*:pCAGGS:GBS-luc:pRL-tk (10:10:10:1).

Figure 3G and S4F (SmoM2+Cdh7 expression assays): *SmoM2*:pCAGGS-*Cdh7*:GBS-luc:pRL-tk (10:10:10:1).

Figure 3H and S4F (Gli3FL expression assays): *Gli3FL*:pCAGGS:GBS-luc:pRL-tk (10:10:10:1).

Figure 3H and S4F (Gli3FL+Cdh7 expression assays); *Gli3FL*:pCAGGS-*Cdh7*:GBS-luc:pRL-tk (10:10:10:1).

Figure S3A,B and S4F (Gli1 or Gli2 expression assays): *Gli1/2*:pCAGGS:GBS-luc:pRL-tk (10:10:10:1).

Figure S3A,B and S4F (Gli1+Cdh7 or Gli2+Cdh7 expression assays): *Gli1/2*:pCAGGS-*Cdh7*:GBS-luc:pRL-tk (10:10:10:1).

Figure S3C and S4F (Gli3R expression assays): *Gli3R*:pCAGGS:GBS-luc:pRL-tk (10:10:10:1).

Figure S3C and S4F (Gli3R+Cdh7 expression assays): *Gli3R*:pCAGGS-*Cdh7*:GBS-luc:pRL-tk (10:10:10:1).

Figure S5A and S5E-J: the expression plasmids (up to three plasmids), the GBS-luc plasmid and the pRL-tk plasmid were electroporated with the ratio of 30(10 for each expression plasmid):10:1. If less than three expression plasmids were used the ratio was adjusted to 30:10:1 using the empty pCAGGS plasmid (control).

Fig. S5C and S5D: the expression plasmid (up to two plasmids), the GBS-luc plasmid and the pRL-tk plasmid were

electroporated with the ratio of 20(10 for each expression plasmid):10:1. If only one expression plasmid was used the ratio was adjusted to 20:10:1 using the empty pCAGGS plasmid (control).

Immunocytochemistry

Detection of Cdh7 aggregation on the cell surface. NIH3T3 cells were cultured on 18 x 18-mm micro cover glasses (Matsunami) in 10-cm diameter tissue culture plates and transiently transfected with pCAGGS-*Cdh7*-6x-myc with or without siRNA-Cdh7 or pCAGGS-*cNcdh* using Lipofectamine 2000 (Invitrogen). The following day, culture media were changed with 10 ml/plate OPTI-MEM (Invitrogen) with or without rN-Shh. After 24 hours, transfected cells were washed using PBS three times and fixed in 4% PFA/PBS for 5 minutes at RT. Cells were incubated with anti-Cdh7 mAb at 4ºC overnight. After incubation with secondary Abs, cells were washed in PBS and incubated with PBS containing Hoechst 33342 for 5 minutes at RT, followed by fluorescence microscope analysis. Quantitative analysis of the distribution of fluorescence intensities in Cdh7-expressing or Ncdh-expressing cells cultured with or without rN-Shh was performed using Adobe Photoshop software.

Detection of Cdh7 association with Sufu. NIH3T3 cells were cultured on 18 x 18-mm micro cover glasses (Matsunami) in 10-cm diameter tissue culture plates and transiently transfected with pCAGGS-*Cdh7* full-length with or without siRNA-Cdh7 or delC, pCAGGS-*cNcdh* or pMES-*Cdh20*-6x myc constructs using Lipofectamine 2000 (Invitrogen). The following day, culture media were replaced with 10 ml/plate OPTI-MEM with or without rN-Shh. After 24 hours, transfected cells were incubated with anti-Cdh7, anti-Ncdh or anti-Cdh20 Abs at 4°C overnight, and fixed in 100% ethanol for 5 minutes at RT. Cells were then permeabilized by washing with PBS containing 0.1% Triton X-100 for 10 minutes and incubated with 5% skim milk containing anti-Sufu pAb. After incubation with secondary Abs, cells were washed in PBS and incubated with PBS containing Hoechst 33342 for 5 minutes at RT, followed by fluorescence microscope analysis.

Detection of Gli3 processing. NIH3T3 cells were cultured on 18 x 18-mm micro cover glasses (Matsunami) in 10-cm diameter tissue culture plates and transiently transfected with pcDNA3.1-GFP-*mGli3*-V5 with or without pCAGGS-*Cdh7* plasmids using Lipofectamine 2000 (Invitrogen). The next day, the culture media were changed with 10 ml/plate OPTI-MEM with or without rN-Shh. After 24 hours, cells were fixed in 4% PFA/PBS for 5 minutes at RT. Cells were permeabilized by

washing with PBS containing 0.1% Triton X-100 for 10 minutes and incubated with 5% skim milk containing anti-GFP mAb and anti-V5 mAb for 1 hour at RT. After incubation with secondary antibodies, cells were washed in PBS and incubated with PBS containing Hoechst 33342 for 5 minutes at RT, followed by fluorescence microscopy analysis.

Detection of Cdh7 and V5-tagged Gli3 peptides. NIH3T3 cells were cultured, transiently transfected with pcDNA3.1-*mGli3*-V5 and pCAGGS-*Cdh7* plasmids and treated with rN-Shh as described above. After 24 hours of rN-Shh treatment, Cdh7-expressing cells were incubated with conditioned medium containing anti-Cdh7 mAb for 4 hours at 4°C and fixed in 4% PFA/PBS for 5 minutes at RT. Cells were then permeabilized and incubated with 5% skim milk containing anti-V5 mAb for 1 hour at RT. After incubation with secondary antibodies, cells were washed in PBS and incubated with PBS containing Hoechst 33342 for 5 minutes at RT.

For all these assays, cells were mounted in PBS containing 90% glycerol and 1% propyl gallate and imaged with a fluorescence microscope (BZ-9000, Keyence or E600 Nikon).

Cell surface images

The Cell surface images were made using Image J and Adobe Photoshop CS extended softwares.

Cell fractionation

NIH3T3 cells were cultured in 10-cm diameter tissue culture plates and transiently transfected with pcDNA3.1-GFP-*mGli3*-V5 or pcDNA3.1-*mGli3*-V5 and pEGFP-N1 plasmids with or without pCAGGS-*Cdh7* plasmid using Lipofectamine 2000. The following day, culture media were changed with 10 ml/plate OPTI-MEM with or without rN-Shh. After 24 hours, cells were fractionated using a ProteExtract Subcellular Proteome Extraction Kit (Calbiochem) to isolate nuclei and cytosol, and 100 µl 2x Laemmli sample buffer were added to each sample. Proteins were resolved by SDS-PAGE, transferred to PVDF membranes, and immunoblotted with the desired antibodies.

Artificial cross-linking assays

NIH3T3 cells were cultured on 18 x 18-mm micro cover glasses (Matsunami) in 10-cm diameter tissue culture plates and transiently transfected with pCAGGS-*Cdh7* plasmid using Lipofectamine 2000. The following day, culture media were replaced with OPTI-MEM. After 24 hours, cells were incubated with conditioned medium (RPMI 1640 with 10% FCS) from hybridoma cells containing anti-Cdh7 mAb or with control conditioned medium (RPMI 1640 with 10% FCS) for 4 hours at RT. Cells were then fixed in 4% PFA/PBS for 5 minutes at RT, followed by permeabilization and incubation with 5% skim milk containing anti-Sufu pAb for 1 hour at RT. After incubation with secondary antibodies, cells were washed in PBS, incubated with PBS containing Hoechst 33342 and imaged by fluorescence microscopy**.** Some of the cells were co-transfected with pCAGGs-*Cdh7*:pCAGGs:GBS-luc:pRL-tk (10:10:10:1 ratio) and assayed following artificial Cdh7 cross-linking using the Dual Luciferase Reporter Assay System to detect RLA.

References for Materials and Methods

Chen Y. X., Krull C. E. and Reneker L. W. (2004). Targeted gene expression in the chicken eye by in ovo electroporation. Mol Vis. 10, 874-883.

Dessaud, E., Yang, L. L., Hill, K., Cox, B., Ulloa, F., Ribeiro, A., Mynett, A., Novitch, B. G., and Briscoe, J. (2007). Interpretation of the sonic hedgehog morphogen gradient by a temporal adaptation mechanism. Nature 450, 717-720.

Hambeurger V., and Hamilton, H. L. (1951). A series of normal stages in the development of chick embryo. J. Morph. 88, 49-92.

Kawano, R., Matsuo, N., Tanaka, H., Nasu, M., Yoshioka, H., and Shirabe, K. (2002). Identification and characterization of a

soluble cadherin-7 isoform produced by alternative splicing. J Biol Chem. 277, 47679–47685.

Megason, S. G. and McMahon, A. P. (2002). A mitogen gradient of dorsal midline wnts organizes growth in the CNS. Development 129, 2087-2098.

Sasaki, H., Hui, C., Nakafuku, M., and Kondoh, H. (1997). A binding site for Gli proteins is essential for HNF-3beta floor plate enhancer activity in transgenics and can respond to Shh *in vitro*. Development 124, 1313-1322.

Schaeren-Wiemers N. and Gerfin-Moser A. (1993). A signal protocol to detect transcripts of various types and expression levels in neural tissue and cultured cells: in situ hybridization using digoxigenin-labelled cRNA probe. Histochemistry. 100, 431-440.

Shirabe, K., Kimura, Y., Matsuo, N., Fukushima, M., Yoshioka, H. and Tanaka, H. (2005). MN-cadherin and its novel variant are transiently expressed in chick embryo spinal cord. Biochem Biophy Res Commun. 334, 108-116.

Williams K. P., Rayhorn, P., Chi-Rosso, G., Garber, E. A., Strauch, K. L., Horan, G. S., Reilly, J. O., Baker, D. P., Taylor, F. R., Koteliansky, V. and Pepinsky, R. B. (1999). Functional antagonists of sonic hedgehog reveal the importance of the N terminus for activity. J Cell Sci. 112, 4405-4414.
